# Regulatory T cells promote sensory neuron growth and protect against neurotoxicity

**DOI:** 10.1016/j.ynpai.2026.100224

**Published:** 2026-06-30

**Authors:** Jessica P. Hayes, Sandra Fok, Renee Whan, Gary D. Housley, Gila Moalem-Taylor

**Affiliations:** a Translational Neuroscience Facility & Department of Physiology, University of New South Wales (UNSW), Sydney, New South Wales, NSW 2052, Australia; b Katharina Gaus Light Microscopy Facility, Mark Wainwright Analytical Centre, Division of Research and Enterprise, University of New South Wales (UNSW), Sydney, New South Wales, NSW 2052, Australia

**Keywords:** Tregs, DRG neurons, Paclitaxel, Chemotherapy-induced peripheral neuropathy

## Abstract

Regulatory T cells (Tregs) maintain immune homeostasis and suppress inflammation, and emerging evidence indicates they also modulate neuronal function and regeneration. Dorsal root ganglion (DRG) sensory neurons transmit pain signals and are damaged by chemotherapeutic agents such as paclitaxel (PTX), leading to neuropathic pain. Although Tregs show therapeutic promise in neuropathic pain, the mechanisms of Treg–neuron interactions remain poorly defined. Using in vitro co-culture, we examined interactions between primary DRG neurons and Tregs and their effect on PTX-induced neurotoxicity. Live-cell imaging showed that activated Tregs enhanced neurite length and branching and preferentially localised to neuron/neurite-rich regions. Additionally, activated Tregs rescued PTX-induced inhibition of neurite outgrowth. Pharmacological blockade identified Treg-derived amphiregulin (AREG) as essential for neurite outgrowth, while both AREG and neuropeptide Y were required for Treg-mediated protection against PTX-induced neurotoxicity. These findings identify Tregs as direct promoters of sensory neuron growth and protection from PTX-induced neurotoxicity through specific molecular mediators, supporting their potential therapeutic relevance in peripheral neuropathic pain.

## Highlights

• Activated Tregs promote neurite outgrowth and branching in DRG sensory neurons.
• Tregs preferentially localise to neuron- and neurite-rich regions in co-cultures.
• Activated Tregs rescue paclitaxel-induced inhibition of neurite outgrowth.
• Treg-derived amphiregulin (AREG) mediates Treg-induced neurite outgrowth.
• Treg-derived AREG and neuropeptide Y mediate protection from PTX neurotoxicity.

## 1 Introduction

Regulatory T cells (Tregs) are a specialised subset of CD4^+^ T cells essential for suppressing inflammation, regulating innate and adaptive immunity, and maintaining homeostasis (Hori et al., 2003; Kawakami and Sakaguchi, 2024; Zou et al., 2026). Tregs suppress excessive immune activation through cytokine secretion, metabolic regulation, and contact-dependent mechanisms, thereby preventing autoimmunity (Sakaguchi et al., 2020; Zou et al., 2026) while supporting tissue repair (Loffredo et al., 2024). Disruption of Treg number or function has been implicated in various autoimmune and inflammatory diseases, including multiple sclerosis, autoimmune gastritis, thyroiditis, type 1 diabetes, allergy, and irritable bowel syndrome (Sakaguchi et al., 2020). Accordingly, Tregs have emerged as promising targets for immunomodulatory therapies, with both preclinical and clinical studies actively investigating their therapeutic potential (Mikami et al., 2025; Wang et al., 2025). Beyond their canonical roles in immune regulation, recent evidence suggests that Tregs exert neuromodulatory effects (Keating et al., 2019), including promoting myelin regeneration (Dombrowski et al., 2017; Duffy et al., 2019), enhancing spinal cord repair (Qin et al., 2025), and modulating peripheral sensory neuron activity (Mendoza et al., 2025). Resident Tregs, typically transcription factor forkhead box P3 (Foxp3) positive, can express several nervous system-specific neuropeptide genes/proteins, such as neuropeptide Y (NPY) (Ito et al., 2019a) and proenkephalin (Aubert et al., 2024; Mendoza et al., 2025; Midavaine et al., 2025; Shime et al., 2020), as well as neuroprotective factors, such as amphiregulin (AREG) (Burzyn et al., 2013; Zaiss et al., 2013), which may promote neural repair.

Dorsal root ganglion (DRG) sensory neurons relay sensory input from the periphery to the central nervous system (CNS) (Nascimento et al., 2018). Damage to these neurons, caused by peripheral nerve injury (PNI) or chemotherapeutic agents like paclitaxel (PTX), can lead to peripheral neuropathic pain, characterised by burning, tingling, electric-shock-like sensations, and hypersensitivity to stimuli (Colloca et al., 2017; Murphy et al., 2020). This condition is driven by DRG neuron hyperexcitability, arising from damaged nerve fibres, immune cell infiltration, and altered gene and protein expression (Esposito et al., 2019). DRG neurons as well as resident and infiltrating immune cells release pro-inflammatory mediators, including neurotrophins, cytokines such as interleukin (IL)-1β, IL-6, and tumour necrosis factor (TNF), interferons, growth factors, chemokines, and adenosine triphosphate (ATP), that further sensitise nociceptive pathways, promote ectopic firing, and sustain chronic pain (Ji et al., 2023; Krames, 2014). Chemotherapy-induced peripheral neuropathy (CIPN) represents a common and debilitating form of neuropathic pain, where agents such as PTX systemically affect all DRG (Liu et al., 2014). PTX, a widely used microtubule-stabilising chemotherapeutic for multiple cancers (e.g. ovarian, breast, and lung cancers, as well as Kaposi's sarcoma) (Alqahtani et al., 2019; Weaver, 2014), accumulates along sensory nerves, damaging axon terminals and DRG cell bodies and inducing dieback degeneration (Chen et al., 2024). In vitro and ex vivo studies have shown that PTX impairs sensory neuron outgrowth, promotes retraction bulb formation and demyelination, and sensitises DRG neurons (Chen et al., 2015; Kawashiri et al., 2018; Livni et al., 2022, Livni et al., 2019; Villalba-Riquelme et al., 2022). Both PNI and PTX-induced peripheral neuropathy are associated with increased infiltration of pro-inflammatory immune cells and reduced anti-inflammatory populations, such as Tregs, resulting in sustained neuroinflammation and persistent neuronal hyperexcitability that maintains pain hypersensitivity (Austin et al., 2012; Chen et al., 2022; Davoli-Ferreira et al., 2020; Jang and Garraway, 2024; Wang et al., 2007).

Although Tregs infiltrate the injury site, DRG, and spinal cord following PNI (Austin et al., 2012; Davoli-Ferreira et al., 2020; Jain et al., 2024; Lees et al., 2015), their numbers are limited. Experimental augmentation of Treg populations via CD28 superagonists, TNF receptor 2 (TNFR2) agonists, intrathecal low-dose IL-2, or adoptive transfer of Tregs, attenuates neuropathic pain behaviours, especially mechanical allodynia, in preclinical models of PNI (Austin et al., 2012; Fischer et al., 2019; Lees et al., 2015; Liu et al., 2014; Midavaine et al., 2025) and PTX-induced pain in mice (Liu et al., 2014). Conversely, Treg depletion has been shown to exacerbate pain (Davoli-Ferreira et al., 2020; Lees et al., 2015). Although the precise mechanisms remain unclear, IL-10 and IL-35 signalling pathways have been implicated in Treg-mediated suppression of neuropathic pain (Davoli-Ferreira et al., 2020; Duffy et al., 2019; Fischer et al., 2019). More recently, intrathecal adoptive transfer of Tregs has been shown to modulate meningeal and peripheral immunity as well as spinal glial responses, resulting in pain reduction in nerve-injured mice (Fiore et al., 2023; Midavaine et al., 2025). However, the direct effects of Tregs on DRG sensory neurons remain underexplored.

Here, we have addressed the direct effects of Tregs on DRG sensory neurons in vitro. We first characterised the interaction between Tregs and DRG neurons using in vitro co-cultures, quantifying neurite outgrowth parameters (total and mean neurite length, complexity, and the number of nodes, neurites, and terminals) and cell-cell attraction using live-cell imaging. We next examined whether Treg treatment mitigates PTX-induced inhibition of neurite outgrowth in DRG sensory neurons in vitro. Given that Tregs can confer neuroprotection through direct contact and soluble mediators (Sakaguchi et al., 2020; Zhang et al., 2017), we focused on the roles of Treg-derived pro-regenerative factors, AREG and NPY.

## 2 Materials and methods

### 2.1 Animals

Female C57BL/6 J and transgenic DEREG (DEpletion of REGulatory T cells) mice were used. DEREG mice express a diphtheria toxin receptor (DTR)-enhanced green fluorescent protein (eGFP) fusion protein under the control of the *Foxp3* promoter (Lahl and Sparwasser, 2011), enabling isolation of eGFP^+^ Tregs using fluorescence-activated cell sorting (FACS). Mice were housed under standard conditions with food and water ad libitum*.* All procedures were approved by the UNSW Animal Care and Ethics Committee (ACEC-19/14B, ACEC-22/5B, ACEC-19/73A, and ACEC-22/74A).

### 2.2 Tregs

#### 2.2.1 Isolation of Tregs

Primary Tregs were isolated from DEREG mice as described in Duffy et al. (2019). Briefly, the lymph nodes and spleen were dissected, mechanically dissociated, subjected to red blood cell lysis, and washed in RPMI-1640 (Invitrogen). The resulting single-cell suspension was incubated in a culture flask at 37 °C with 5% CO_2_ for 1 h to allow monocyte adherence. The non-adherent cell fraction was transferred to a 15 mL tube and centrifuged at 300 x*g* for 5 min at 25 °C. After discarding the supernatant, cells were resuspended in complete Hanks' Balanced Salt Solution (HBSS) (HBSS [Gibco] + 0.5% FBS [ThermoFisher] + 20 mM HEPES [Gibco] + 1% penicillin-streptomycin [PS; ThermoFisher]). Foxp3/GFP^+^ Tregs were isolated by FACS using a FACSAria™ III (BD Biosciences) or FACSMelody™ (BD Biosciences) cell sorter and collected into a 15 mL tube containing complete HBSS.

#### 2.2.2 In vitro activation of Tregs

Sorted Tregs were centrifuged at 500 ×*g* for 3 min at 25 °C and resuspended in complete Treg medium (RPMI-1640 supplemented with 5% FBS, 10 mM HEPES, 1% non-essential amino acids [Gibco], 1% sodium pyruvate [Gibco], 0.1% β-mercaptoethanol [Gibco], and 1% penicillin–streptomycin). Cells were counted using a Countess II Automated Cell Counter (Invitrogen), then centrifuged again at 600 ×*g* for 3 min at 25 °C. The pellet was resuspended in complete Treg medium and divided into activated and resting culture conditions. For the activated condition, Tregs were stimulated with 50 ng/mL recombinant IL-2 (R&D Systems) and CD3/CD28 Dynabeads (ThermoFisher) at a 1: 1 cell-to-bead ratio, as previously described (Duffy et al., 2019). For the resting condition, Tregs were cultured in complete Treg medium without additional stimulation. Cells were seeded at 1 × 10^5^ cells in 200 μL per well in a 96-well plate and maintained under their respective culture conditions for 7 days at 37 °C and 5% CO₂ prior to use in subsequent experiments.

### 2.3 Dissociation of DRG sensory neurons

DRG neurons were dissociated from C57BL/6 J mice as described by Seong-il et al. (2015). Briefly, DRGs were aseptically dissected and enzymatically dissociated in Neurobasal Medium A (NBM-A; Gibco) containing 10 μg/mL collagenase (ThermoFisher) for 1 h at 37 °C, followed by 0.25% Trypsin-EDTA (ThermoFisher) for 15 min. The tissue was then mechanically dissociated in culture medium (NBM-A supplemented with 2% B-27 [Gibco] and 1% penicillin–streptomycin) using gentle trituration and filtered through a 70 μm strainer. The resulting cell suspension was centrifuged, resuspended in L-15 medium (ThermoFisher), layered over a pre-prepared 30–60% Percoll gradient, and centrifuged again. Cells at the interphase were collected and washed twice by resuspension in L-15 medium followed by centrifugation.

Neurons were finally resuspended in complete neuronal medium (NBM-A supplemented with 2% B-27 [Gibco], 1% GlutaMax [Gibco], and 1% penicillin–streptomycin) and seeded at either 8000–10,000 neurons per 5 μL into the centre of each well of a 96-well plate for the fixed-cell imaging and co-culture live-cell imaging experiments, or 30,000 neurons per 35 μL into the centre of 35 mm glass-bottom FluroDish plates for the monoculture live-cell imaging experiments. All plates were pre-coated with poly-d-lysine (PDL; Sigma Aldrich) and laminin (ThermoFisher) prior to seeding. Neurons were incubated for 30 min at 37 °C with 5% CO₂ to allow adherence, after which each well was gently flooded with 200 μL of complete neuronal medium. Cultures were incubated overnight at 37 °C with 5% CO_2_.

### 2.4 Preparation of DRG neuron and Treg co-cultures for live cell imaging

Tregs were mechanically aspirated from each well, and the CD3/CD28 Dynabeads were removed using a Dynal magnetic separation rack (Invitrogen). Cells were centrifuged, resuspended in complete neuronal medium containing 1.5× CellMask™ Deep Red (ThermoFisher), and incubated for 15 min at 37 °C and 5% CO_2_. Stained Tregs were then washed twice by centrifugation and resuspension in complete neuronal medium.

**Fig. 1:**
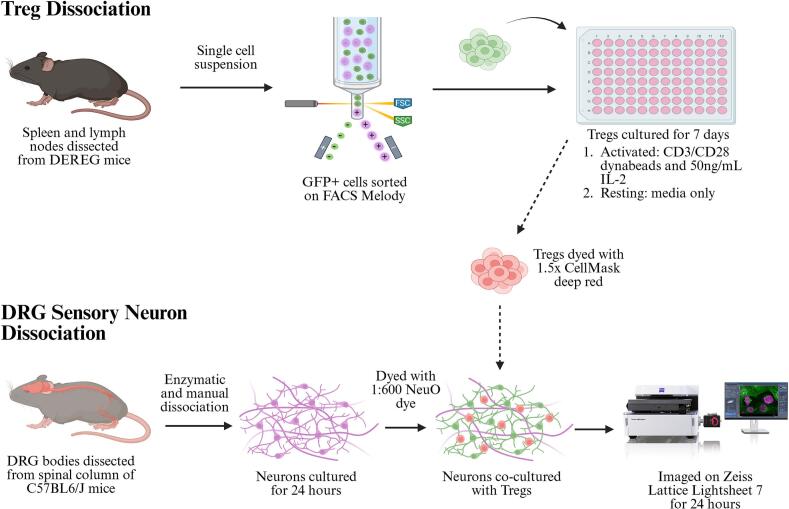
**DRG sensory neuron and Treg dissociation and co-culture timeline.** Flow chart of dissociation, co-culturing, and imaging process for DRG sensory neurons and Tregs. Created in BioRender. License: Moalem-Taylor, G. (2026) https://BioRender.com/y2ct3vo.

DRG neuron cultures were stained with 1:600 NeuO dye (StemCell Technologies) in complete neuronal medium for 1 h, after which the wells were washed twice with fresh medium. Neurons were co-cultured with either CellMask™-labelled resting or activated Tregs at approximately 1 × 10^5^ Tregs per well, with neuron-only cultures included as negative controls. Co-cultures were imaged every 2 h for 24 h using the ZEISS Lattice Lightsheet 7 microscope, with an illumination objective lens 13.3× / 0.4 and a detection objective lens 44.83× / 1.0, using a 100 μm × 1400 mm lightsheet, with a step size of 200 nm. Laser line 488 nm was used to excite DRG neurons stained with NeuO, and 640 nm was used to excite Tregs labelled with CellMaskTM Deep Red. For more details on image processing, refer to the *Live-cell imaging (ZEISS Lattice Lightsheet 7)* methodology section and **Table S1**. The experimental design and timeline are illustrated in Fig. 1.

### 2.5 Drug preparation for culture treatment

PTX powder (In Vitro Technologies) was reconstituted in dimethyl sulfoxide (DMSO) to a 10 mM stock solution. For culture treatments, the stock was diluted in complete neuronal media to final concentrations of 100 nM or 1 μM.

AG1478 (Sigma-Aldrich), an AREG receptor antagonist, and BIBO3304 (Sigma-Aldrich), a NPY1 receptor antagonist, were reconstituted in DMSO to 10 mg/mL and 2 mg/mL stock solutions, respectively. For culture treatments, the stock solutions were diluted in complete neuronal media to final concentrations of 10 nM, 100 nM, or 1 μM.

### 2.6 PTX treatment of DRG neuron cultures

In experiments assessing the effects of PTX on sensory neurons using live-cell imaging, immediately prior to imaging, neurons were stained with NeuO dye, as described above. The cells were then treated with either 100 nM PTX or vehicle control, and monocultures were imaged every 20 min over 24 h using the ZEISS Lattice Lightsheet 7 microscope.

In experiments assessing the effects of Tregs on PTX-treated neurons, 24 h after seeding, DRG neuron cultures were treated with 1 μM PTX or vehicle for 12 h. Following treatment, the neurons were washed, co-cultured with 100,000 activated Tregs per well and treated with vehicle, AG1478 or BIBO3304. Cultures were incubated for an additional 12 h before fixation with 4% paraformaldehyde (PFA), and neurite outgrowth was assessed by immunocytochemistry. A greater PTX treatment concentration was used in these experiments, as neurite outgrowth recovered in the washout and co-culture period following treatment with only 100 nM PTX (**Fig. S1**).

### 2.7 Immunocytochemistry of DRG sensory neuron cultures

Fixed DRG neuron cultures were permeabilised with 0.1% Triton X-100 in PBS and then blocked with PBS containing 0.1% Tween-20 (PBS-T) and 5% normal donkey serum (NDS; Sigma Aldrich) for 30 min at RT. Primary staining was performed using mouse anti-mouse β-III-tubulin (neuronal marker, 1:1000; Sigma-Aldrich) in PBS-T containing 2% NDS and incubation for 1 h at 4 °C. After three PBS washes, cells were incubated with Alexa-Fluor conjugated donkey anti-mouse 488 or 647 secondary antibody (1:1000; Life Technologies) in PBS-T containing 2% NDS for 1 h at RT in the dark. Following three PBS washes, nuclei were stained with Hoechst 33342 stain (1:10,000; Life Technologies) in PBS for 15 min in the dark and washed twice with PBS. Cultures were stored in PBS containing 1% sodium azide at 4 °C in the dark until imaging. Tregs were not stained, as they were non-adherent and removed during the immunostaining process.

### 2.8 Imaging

#### 2.8.1 Live-cell imaging (ZEISS lattice Lightsheet 7)

Culture plates were transferred to the ZEISS Lattice Lightsheet 7 microscope incubation chamber for volumetric imaging of subcellular structures and dynamic cellular interactions. Image acquisition was performed every 20 min for 24 h for monocultures and every 2 h for 24 h for co-cultures. The imaging parameters are detailed in **Table S1**. Neurons meeting predefined inclusion criteria (e.g., healthy morphology and clearly identifiable neurites) were randomly selected for imaging by an experimenter blinded to treatment group.

Image processing was conducted using ZEN software (versions 3.2–3.4). Raw images were first deskewed via linear interpolation using the cover glass transformation method, which corrects the misalignment of adjacent *Z*-planes created by the angled orientation of the detection objective relative to the stage movement axis. Maximum intensity projection was subsequently applied to reduce the file size from approximately one terabyte to 3 gigabytes, facilitating efficient data handling and analysis. Deconvolution was applied when enhanced resolution was required. Processed image stacks were then imported into Neurolucida 360 (version 2021.1.3) for quantitative analysis. A representative timelapse video of DRG sensory neurons co-cultured with activated Tregs is available in **Video S1**.

#### 2.8.2 Imaging of fixed neuronal cultures (ZEISS LSM 710 intravital multiphoton/confocal microscope)

Fixed neuron cultures were imaged using the ZEISS LSM 710 intravital multiphoton microscope. Alexa Fluor 647- and Alexa Fluor 488-labelled β-III tubulin were detected using the MaiTai HP pulsed infrared laser (633 nm) and LASOS argon ion laser (454–514 nm), respectively. Images were acquired in 8-bit depth with a resolution of 4096 × 4096 pixels and a pixel area of 0.42 μm^2^. The entire neuronal seeding area was imaged using a 5× objective without tiling.

### 2.9 Image analysis

#### 2.9.1 Neuron summary and cell body contour analyses with NeuroLucida 360

Images acquired from 0, 4, 8, 12 and 16 h from co-cultures and 0, 200, 400, 600 and 800 min from monocultures were analysed. The membrane-permeable dyes used in this study were gradually metabolised by the cells, leading to loss of signal intensity beyond 16 h, which precluded reliable image analysis at later time points. This can be observed in **Video S1**.

Image analysis was conducted using NeuroLucida 360 (version 2021.1.3). Neuron somas were traced using the Auto Contour function, with each cell body assigned a unique colour. Neurites were traced semi-automatically in the 360 3D Environment using the User-Guided Trace Tree tool. Traced neurons within each image were grouped into a ‘SET’ (**Fig. S2**), and analyses were performed using NeuroLucida Explorer (version 2020.1.1) with the Branched Structure Analysis module. The neurite architecture of each neuron was quantified as total neurite length, mean neurite length, complexity, and number of nodes, terminals and neurites (Table 1) using the inbuilt Neuron Summary analysis feature. The cell body contour analysis feature was also used to measure neuron soma morphology by quantifying the soma perimeter, area, feret maximum, feret minimum, aspect ratio, compactness, convexity, form factor, roundness and solidity (Table 1).

**Table 1:** Definition of parameters measured in neuron summary and cell body contour analyses from NeuroLucida 360.

Parameter	Definition
***Neuron Summary Analysis***	
Number of neurites	Number of processes extending from neuron soma
Number of nodes	Number of branch points per neuron
Number of terminals	Number of branch end points per neuron
Total neurite length	Total length of all branches in the neuron tree
Mean neurite length	= [Length] / [Number of branches]
Neurite complexity	= [Σ Terminal Orders + Number of Terminals] × [Total Neurite Length / Number of Neurites] Terminal order refers to the number of branch points encountered when tracing from the terminal end of a neurite to the soma.
***Cell Body Contour Analysis***	
Perimeter	Length of contour representing the cell body
Area	The cross-sectional area contained within the boundary of the cell body
Feret maximum	Largest distance between any two boundary points of the cell contour
Feret minimum	Smallest distance between any two boundary points of the cell contour
Aspect ratio	Feret maximum / Feret minimum
Compactness	√(4/π × Area) / Maximum Diameter
Convexity	Convex Perimeter / Perimeter
Form factor	(4π × Area) / (Perimeter)^2^. A measure of perimeter complexity.
Roundness	(Compactness)^2^
Solidity	Area / Convex Area

#### 2.9.2 Treg density analysis

Timelapse images were exported to ImageJ for Treg density analysis. A grid overlay with a fixed area of 6110 μm^2^ per square was applied to each image, and the number of Tregs within each grid square was manually counted using the Marker tool. Each grid square (or quadrant) was categorised into one of two conditions: (1) quadrants containing neurons, and (2) quadrants not containing neurons. Quadrants were assigned to a condition based on the criteria outlined in **Table S2** and illustrated in Fig. 5A.

Treg density was calculated as the number of Tregs per imaging quadrant (6110 μm^2^). For quadrants containing a DRG neuron soma, the soma area was subtracted from the total quadrant area (6110 μm^2^) to account for space unavailable to Tregs, and density was calculated relative to the remaining accessible area.

#### 2.9.3 Analysis of Treg association with DRG sensory neuron terminals

NeuroLucida 360 was used to assess the association of Tregs with DRG sensory neuron terminals at the final analysis time point (16 h). Neurite endpoints and Tregs were marked in the 3D Environment window using the marking feature (Fig. 6B). A locus analysis was performed in NeuroLucida Explorer, which calculated the straight-line distance between each neurite endpoint marker and each Treg marker. Tregs located within a 25 μm radius of a neurite terminal were counted, and this number was divided by the total number of neurite terminals to yield the number of Tregs per endpoint as a measure of association. A 25 μm distance was selected based on prior evidence suggesting that this proximity facilitates Treg-neuron communication via either contact-dependent mechanisms or short-range soluble mediators (Mendoza et al., 2025).

To determine if proliferation influenced association, the density of Tregs per image at the 16-h timepoint was calculated. Tregs were manually counted in each image using the marking tool FIJI ImageJ. As the image dimensions varied between imaging setups, the total number of Tregs per image was normalised to image area, yielding a Treg density value for each image.

#### 2.9.4 Neurite outgrowth analysis from fixed cell imaging

Confocal images were analysed using FIJI (Image J version 1.54j; Bethesda, MD, USA) to quantify neurite outgrowth, measured as membrane surface area. Images were threshold adjusted, and the integrated density (i.e. the sum of all pixel intensities) was calculated. The number of pixels representing the neurite membrane was determined by dividing the integrated density by 255 (the maximum pixel value for 8-bit images). The value was then multiplied by pixel area (0.42 μm^2^) to determine the membrane surface area.

Due to inter-experimental variability in neurite outgrowth, membrane surface area values were normalised to the mean of each biological replicate prior to analysis.

### 2.10 Statistical analysis

Statistical analyses were performed using GraphPad Prism 10 and Estimation Statistics. For live-cell imaging, neuron summary and cell body contour data were normalised to the 0-h timepoint and analysed across 4-, 8-, 12- and 16-h timepoints for co-cultures and 200, 400, 600, and 800 min for monocultures. The Shapiro-Wilk test confirmed normal distribution of all datasets, allowing for analysis with repeated-measures two-way ANOVA or mixed-effects ANOVA, followed by Tukey's post hoc test for multiple comparisons. For fixed cell imaging, neurite outgrowth data were analysed by one-way ANOVA and Tukey's post hoc test or two-stage step-up method of Benjamini, Krieger, and Yekutieli to control the false discovery rate.

Estimation statistics were also applied to neuron summary data to assess effect sizes across treatment conditions using the open-source platform estimationstats.com. This approach reports effect sizes with confidence intervals (CIs) rather than relying solely on null-hypothesis significance testing. Hedge's g was calculated for repeated-measures comparisons at each time point and interpreted as small (*g* < 0.5), medium (0.5 < *g* < 0.8), large (0.8 < *g* < 1.2), or very large (*g* > 1.2) (Brydges et al., 2019; Fiore et al., 2024). Medium, large and very large effect sizes and their corresponding 95% CIs are reported. Positive *g* values indicate an increase and negative *g* values a decrease in the measured parameter. 95% CIs were generated using 5000 bootstrap resamples.

Treg density data were grouped into 0–4, 8, and 12–16-h intervals due to the absence of significant differences between 0- and 4-h, and 12- and 16-h time points. A mixed-effects model with Tukey's post hoc test was used to compare conditions at each time point. Unpaired two-tailed *t*-tests were used to analyse the number of Tregs per neurite terminal and Treg density per image. A simple linear regression assessed the relationship between Treg proliferation and their association with neurite terminals in activated versus resting Treg + neuron co-cultures.

Data are presented as violin plots to illustrate the distribution. Statistical significance is denoted with asterisks (*) and effect size with hash symbols (#).

## 3 Results

### 3.1 Regulatory T cells enhance DRG sensory neuron outgrowth, but do not influence their soma morphology, in vitro

**Fig. 2:**
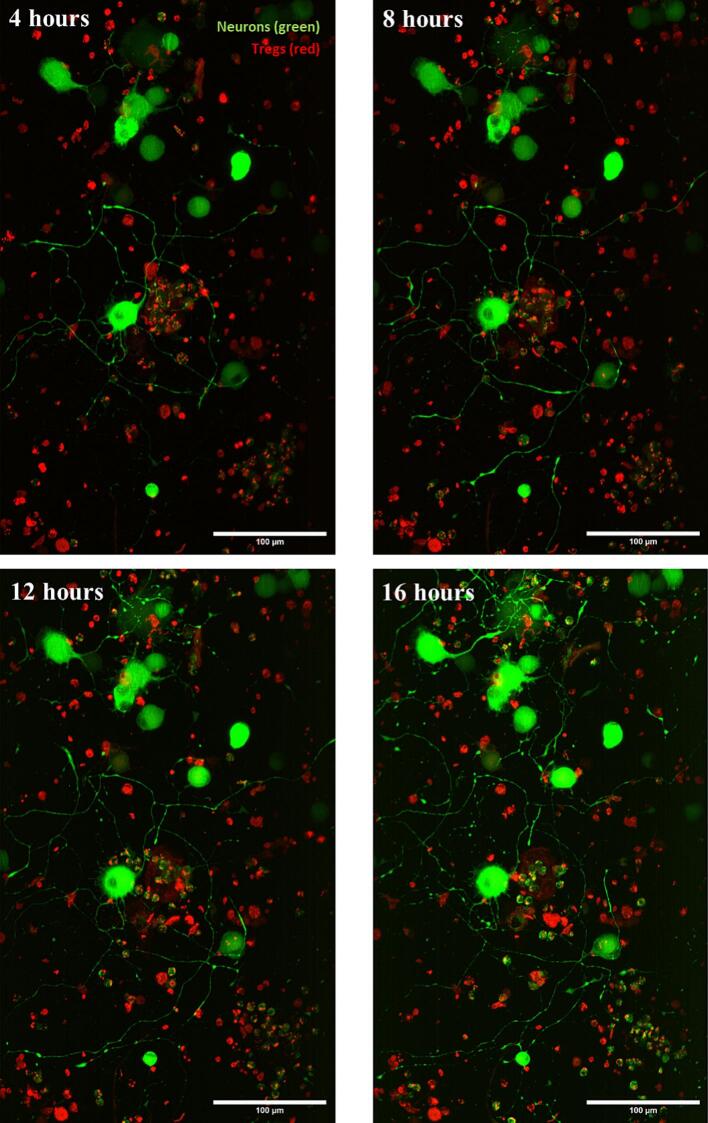
**Representative images of DRG sensory neuron and activated Treg co-culture at 4-, 8-, 12- and 16-h time points.** DRG sensory neurons were grown for 24 h before being co-cultured with Tregs and imaged every 2 h for 24 h on the ZEISS Lattice Lightsheet 7 microscope. Neurons are dyed with NeuO dye (green), and Tregs are dyed with CellMask™ deep red (red). Scale bar represents 100 μm. (For interpretation of the references to colour in this figure legend, the reader is referred to the web version of this article.)

To investigate the effects of Tregs on neuronal outgrowth, DRG sensory neurons were co-cultured with primary activated Tregs (stimulated ex vivo with anti-CD3/CD28 and IL-2), or primary resting Tregs, and imaged over a 24-h period using a ZEISS Lattice Lightsheet 7 microscope (Fig. 1**)**. Neurite outgrowth was assessed across several parameters: total and mean neurite outgrowth, complexity, and number of nodes, terminals and neurites. Representative timelapse images of neurons co-cultured with activated Tregs are shown in Fig. 2, with a corresponding timelapse video available in **Video S1**. Representative images of DRG sensory neuron and resting Treg co-culture are shown in **Fig. S3**.

**Fig. 3:**
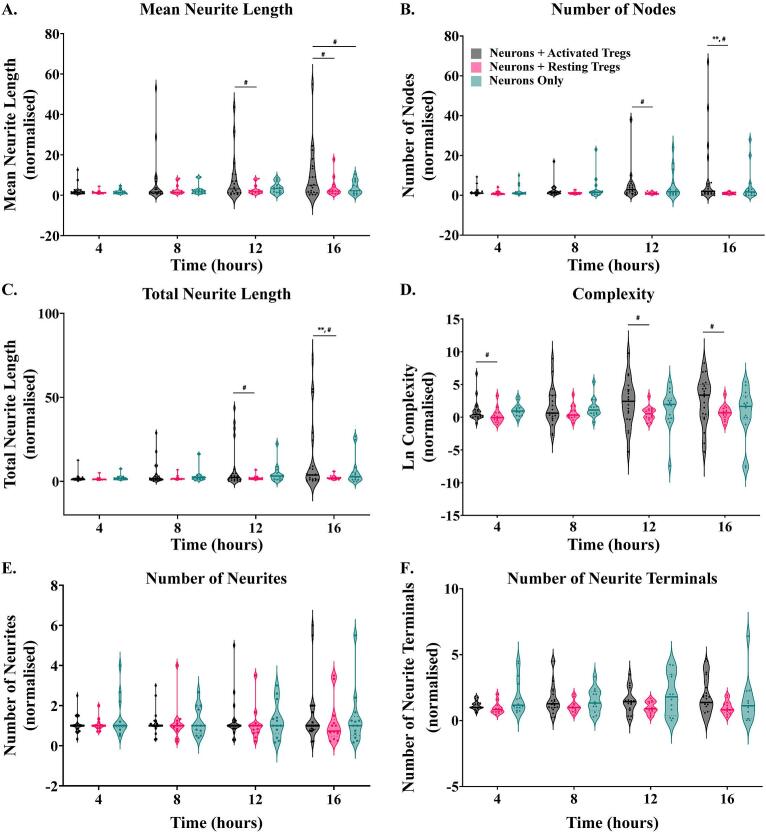
**Activated Tregs facilitate neurite outgrowth of DRG sensory neurons in co-culture**. DRG sensory neurons were cultured alone (teal, *n* = 6 animals from 3 independent DRG cell dissociations with 2 animals pooled per dissociation; 15 neurons), with resting Tregs (pink *n* = 12 animals from 3 independent DRG cell dissociations and 3 independent Treg dissociations with 2 animals pooled per dissociation; 15 neurons), or with activated Tregs (black, *n* = 6 animals from 3 independent DRG cell dissociations and 3 independent Treg dissociations with 2 animals pooled per dissociation; 21 neurons). Images were acquired every 2 h for 24 h; data from 4, 8, 12, and 16 h were analysed and normalised to baseline (*t* = 0 h). Complexity values (panel D) were log-transformed (natural log). **(A)** Mean neurite length (μm), **(B)** number of nodes, **(C)** total neurite length (μm), **(D)** ln complexity, **(E)** number of neurites, and **(F)** number of neurite terminals. Statistical analysis was performed using two-way ANOVAs or mixed-effects models with multiple comparisons and estimation statistics for effect size. Data are presented as violin plots with median lines. **p < 0.001 and # for medium effect size (0.5 < *g* < 0.8). (For interpretation of the references to colour in this figure legend, the reader is referred to the web version of this article.)

We found a significant main effect of culture condition on mean neurite length (*F* (2, 137) = 5.300, *p* = 0.0061; Fig. 3A) and number of nodes (*F* (2, 175) = 3.900, *p* = 0.0220; Fig. 3B) of DRG sensory neurons. We also observed a significant main effect of time on total neurite length, indicating that neurite length increased over time across culture conditions (*F* (2,102) = 3.137, *p* = 0.0476; Fig. 3C). Post-hoc comparisons revealed that cultures containing activated Tregs exhibited greater total neurite length (*p* = 0.0062; Fig. 3C) and a higher number of nodes (*p* = 0.0049; Fig. 3B) at 16 h compared with cultures containing resting Tregs. These results suggest that activated Tregs promoted outgrowth and branching in DRG sensory neurons.

Estimation statistics comparing neurons co-cultured with activated Tregs relative to neurons with resting Tregs or neurons alone corroborated these findings, with medium effect sizes (0.5 < g < 0.8) for total neurite length, mean neurite length, complexity, and number of nodes **(**Fig. 3A-D**)**. Activated Treg + neuron co-cultures increased mean neurite length at 12 h (*g* = 0.565, [95.0% CI 0.0633, 0.898]) and 16 h (*g* = 0.573 [95% CI -0.114, 0.942]) **(**Fig. 3A**)**, number of nodes at 12 h (*g* = 0.567, [95.0% CI 0.238, 0.76]) and 16 h (*g* = 0.6, [95.0%CI 0.358, 0.867]) **(**Fig. 3B**)**, total neurite length at 12 h (*g* = 0.587 [95.0% CI 0.244, 0.942]) and 16 h (*g* = 0.668 [95.0% CI 0.37, 0.981]) **(**Fig. 3C**)**, and complexity at 4 h (*g* = 0.53, [95.0% CI -0.262, 1.03]), 12 h (*g* = 0.584, [95.0% CI -0.117, 1.2]) and 16 h (*g* = 0.595, [95.0% CI -0.0881, 1.26]) **(**Fig. 3D**)** relative to resting Treg + neuron co-cultures. Activated Treg + neuron co-cultures also increased mean neurite length compared to neuron-only cultures at 16 h (*g* = 0.58, [95.0% CI 0.0503, 0.93]) **(**Fig. 3A**)**.

There were no differences between the conditions for the number of neurites **(**Fig. 3E**)** and the number of neurite terminals **(**Fig. 3F**)**.

**Fig. 4:**
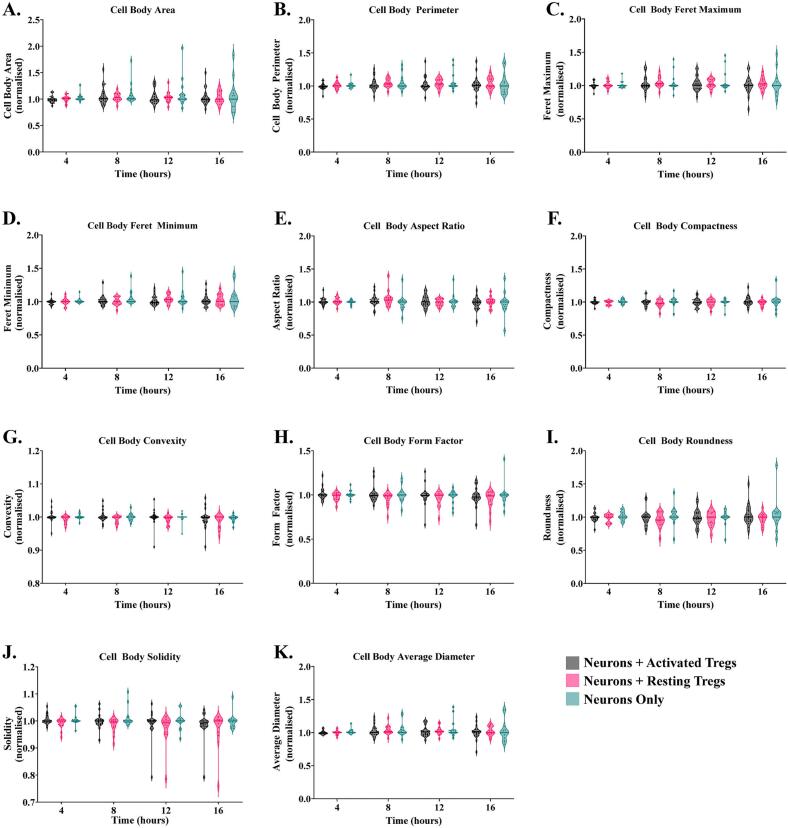
**Tregs do not alter DRG sensory neuron soma morphology in co-culture.** DRG sensory neurons were cultured alone (teal, *n* = 6 animals from 3 independent DRG cell dissociations with 2 animals pooled per dissociation;15 neurons), with resting Tregs (pink, *n* = 12 animals from 3 independent DRG cell dissociations and 3 independent Treg dissociations with 2 animals pooled per dissociation; 15 neurons), or with activated Tregs (black, *n* = 12 animals from 3 independent DRG cell dissociations and 3 independent Treg dissociations with 2 animals pooled per dissociation; 21 neurons). Images were acquired every 2 h for 24 h; data from 4, 8, 12, and 16 h were analysed and normalised to baseline (t = 0 hs). Soma morphology parameters quantified included **(A)** area (μm^2^), **(B)** perimeter (μm), **(C)** Feret maximum (μm), **(D)** Feret minimum (μm), **(E)** aspect ratio, **(F)** compactness, **(G)** convexity, **(H)** form factor, **(I)** roundness, **(J)** solidity, and **(K)** average diameter (μm). Statistical analysis was performed using two-way ANOVAs or mixed-effects models and data are presented as violin plots with median lines. (For interpretation of the references to colour in this figure legend, the reader is referred to the web version of this article.)

We also assessed the effects of Tregs on DRG sensory neuron soma morphology by quantifying soma perimeter, area, Feret maximum, Feret minimum, aspect ratio, compactness, convexity, form factor, roundness, solidity, and average diameter of the neuron somas across timepoints. Mixed-effects analysis revealed significant main effects of time for several soma morphology parameters, including soma area (*F* (1.976, 92.87) = 4.160, *p* = 0.0190; Fig. 4A), perimeter (*F* (2.114, 100.1) = 4.617, *p* = 0.0108; Fig. 4B), Feret maximum (*F* (2.207, 103.7) = 3.510, *p* = 0.0293; Fig. 4C), Feret minimum (*F* (2.400, 114.4) = 3.759, *p* = 0.0196; Fig. 4D), solidity (*F* (1.294, 60.37) = 4.493, *p* = 0.0289; Fig. 4J**)**, and average diameter (*F* (2.155, 101.3) = 4.422, *p* = 0.0125; Fig. 4K**)**, indicating time-dependent changes in several aspects of DRG soma morphology. No significant main effects of time were found for the other cell body parameters: aspect ratio, compactness, convexity, form factor and roundness (Figs. 4E-I, respectively). Furthermore, no significant main effects of co-culture condition or time x condition interactions were observed for any parameter, indicating that Treg co-culture does not significantly affect neuronal soma morphology under these conditions.

### 3.2 Bidirectional attraction between activated Tregs and DRG sensory neurons in vitro

**Fig. 5:**
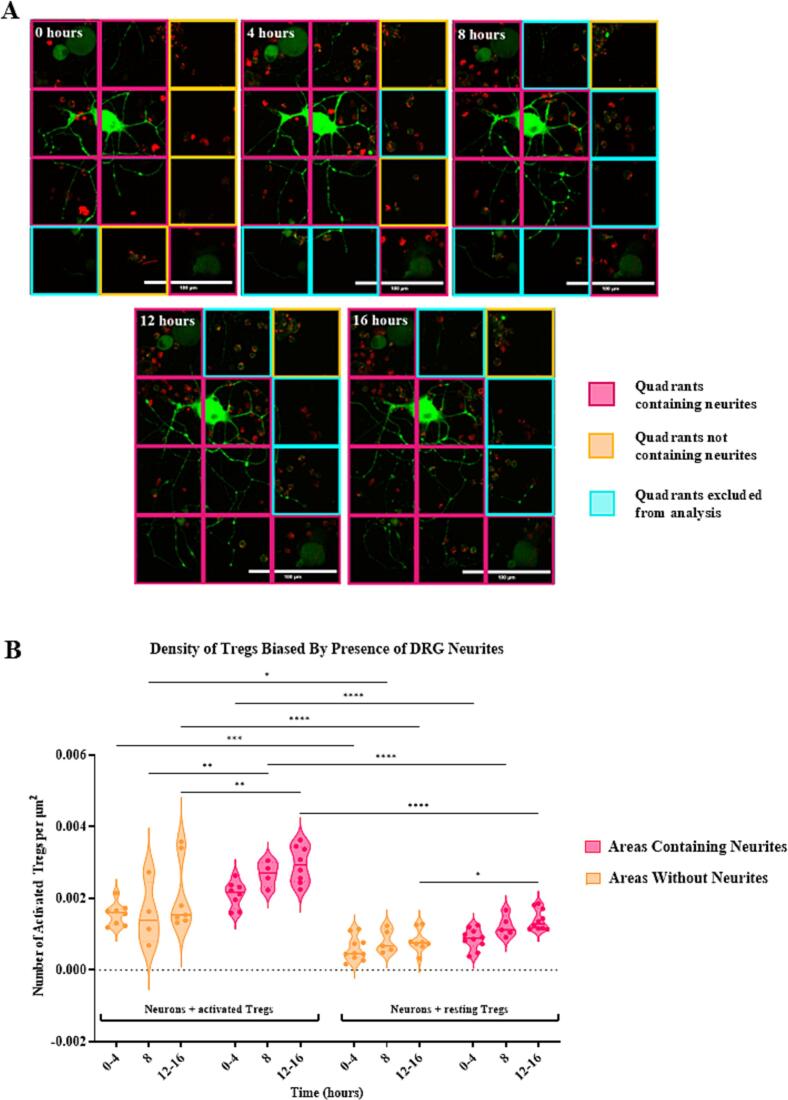
**Tregs preferentially localise to neurite-rich areas in DRG co-cultures. (A)** Representative images of activated Treg and DRG sensory neuron co-cultures across analysed time points (0, 4, 8, 12, and 16 h), with a 6100 μm^2^ grid overlay. Neurons are dyed with NeuO dye (green), and Tregs are dyed with CellMask™ deep red (red). The grid overlay has been colour-coded to represent the different quadrant conditions: Pink boxes represent quadrants that contain neurons/neurites; gold boxes represent quadrants that do not contain neurons/neurites; and blue boxes represent quadrants that were excluded from the analysis as neither Tregs nor neurites were present, or only small segments of neurites had entered the quadrant. Scale bars represent 100 μm. **(B)** Treg density was quantified at 0–4 h, 8 h, and 12–16 h in neuron + activated Treg co-cultures (left) and neuron + resting Treg co-cultures (right) in areas containing neurites (pink) and areas without neurites (gold). *n* = 12 animals from 3 independent DRG cell dissociations and 3 independent Treg dissociations with 2 animals pooled per dissociation; 3–5 wells. Statistical analysis was performed using a mixed-effects model with multiple comparisons. Data are presented as violin plots with median lines * *p* < 0.05, ** *p* < 0.01, *** *p* < 0.001, **** *p* < 0.0001. (For interpretation of the references to colour in this figure legend, the reader is referred to the web version of this article.)

To measure the attraction of Tregs to DRG sensory neurons, Treg density was quantified over time in areas with or without neurites in both activated and resting Treg + neuron co-cultures. Representative images of quadrant analysis are presented in Fig. 5A. Mixed-effects analysis revealed significant main effects of time (*F* (2, 43) = 30.99, *p* < 0.0001) and quadrant condition (*F* (3,32) = 33.02, *p* < 0.0001) on Treg density, with no significant interaction, indicating that time and spatial proximity to neurites independently influenced Treg distribution **(**Fig. 5B**)**. Multiple comparisons testing found that in activated Treg + neuron co-cultures, Treg density was significantly greater in neurite-containing regions than in neurite-free regions at both 8 h (*p* = 0.0013) and 12–16 h (*p* = 0.0020). A similar, though less pronounced, pattern was observed in resting Treg + neuron co-cultures, where Treg density was higher in neurite-containing regions at 12–16 h (*p* = 0.0326) **(**Fig. 5B**)**. These findings suggest that both activated and resting Tregs are progressively attracted to neuronal regions over time, as visualised in Fig. 5A.

Furthermore, activated Tregs were consistently more abundant than resting Tregs across all timepoints, regardless of spatial proximity. Treg density was significantly higher in activated Treg + neuron co-cultures than in resting Treg + neuron co-cultures within both neurite-containing areas (all *p* < 0.0001) and neurite-free areas (0–4 h: *p* = 0.0001, 8 h: *p* = 0.0201, 12–16 h: *p* < 0.0001) **(**Fig. 5B**)**.

**Fig. 6:**
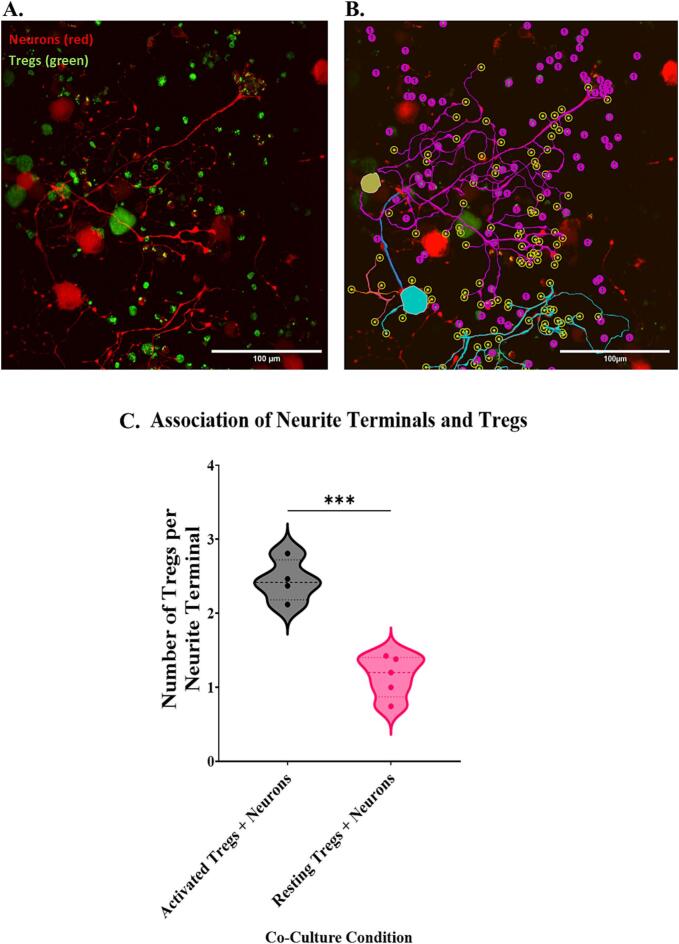
**Activated Tregs are strongly attracted to DRG sensory neuron terminals. (A)** Representative image of an activated Treg (green) + DRG sensory neuron (red) co-culture acquired at 16 h using the ZEISS Lattice Lightsheet 7 microscope. Scale bar = 100 μm. Note that cell colours are reversed compared to Fig. 1 due to an inadvertent discrepancy introduced during the artificial colouring process used for image tracing. **(B)** Corresponding traced image of **(A).** Neuron somas (solid yellow and blue) were traced using the *Autocontour* function in NeuroLucida 360, and neurites (magenta, blue, peach, orange) were traced semi-automatically using *User-Guided Trace Tree* in the 3D Environment. Neurite terminals are marked with yellow circles containing a ‘+’ symbol, and Tregs are marked with magenta circles containing a ‘1’. Straight-line distances between Treg and terminal markers were measured using the *Locus Analysis* tool in NeuroLucida Explorer. Scale bar = 100 μm. **(C)** Treg attraction to neurite terminals was assessed by counting the number of Tregs within 25 μm of each terminal at 16 h and calculating the number of Tregs per terminal. Data represent the mean number of Tregs per neurite across 3–8 neurons per culture. *n* = 6 animals from 3 independent DRG cell dissociations with 2 animals pooled per dissociation; 4–5 wells, 3–8 neurons per image. Statistical analysis was performed using an unpaired two-tailed *t*-test. Data are presented as violin plots with median and quartile lines. *** *p* = 0.0003. (For interpretation of the references to colour in this figure legend, the reader is referred to the web version of this article.)

As sensory nerve terminals are critical sites for signal transduction (Reichardt & Kelly, 1983; Steinhoff et al., 2022), we next investigated whether Tregs preferentially localise near these regions. To assess this, the number of Tregs within a 25 μm radius of each neurite terminal was quantified at the final timepoint (16 h) in both co-culture conditions. This distance supports the potential for contact-dependent interactions or communication via short-range secreted factors (Mendoza et al., 2025). Treg association was calculated as the number of Tregs per neurite terminal. A representative image of an activated Treg + neuron co-culture and its corresponding NeuroLucida 360 trace are shown in Fig. 6A-B.

We found that there was a significantly greater Treg association with neurite terminals in activated Treg + neuron co-cultures compared with resting Treg + neuron co-cultures (*t* (7) = 6.783, *p* = 0.0003; Fig. 6C). Treg density per image was also significantly higher in the activated condition (*t* (7) = 2.410, *p* = 0.0468) **(Fig. S4A)**, potentially reflecting enhanced proliferation of activated Tregs after 16 h in co-culture. However, the magnitude of Treg association with neurite terminals (approximately two-fold higher in the activated co-cultures) suggests that increased proximity cannot be explained by proliferation alone. Supporting this, a simple linear regression revealed a weak, non-significant correlation between Treg-neurite terminal association (number of Tregs per neurite terminal) and overall Treg density (*r*^2^ = 0.2965; *F* (1, 7) = 2.951, *p* = 0.1295; **Fig. S4B**).

Together, these results indicate that activated Tregs are more strongly attracted to DRG sensory neuron terminals than resting Tregs, and that this association is likely driven by directed cellular interactions rather than proliferation.

### 3.3 PTX rapidly inhibits multiple parameters of DRG sensory neuron outgrowth

**Fig. 7:**
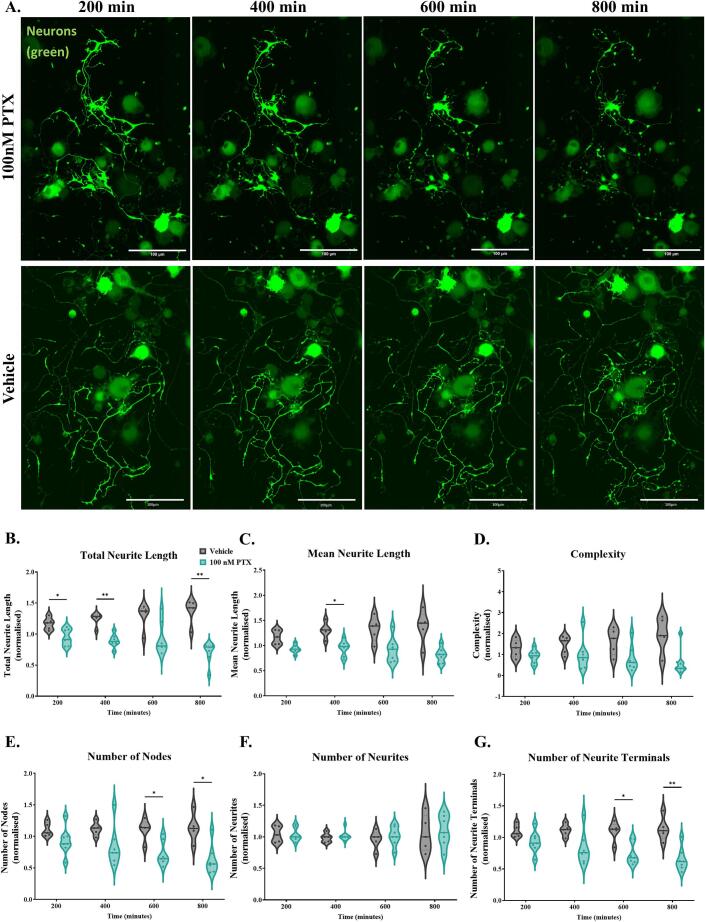
**PTX induces early and sustained inhibition of DRG neurite outgrowth. (A**) Representative images of DRG sensory neurons treated with 100 nM PTX (top row) or vehicle (bottom row) at 0-, 200-, 400-, 600-, and 800 min. Neurons were labelled with NeuO dye (green). Scale bar represents 100 μm. **(B-G)** Quantification of neurite outgrowth over 800 min in vehicle-treated (black, *n* = 6 animals from 3 independent DRG cell dissociations with 2 animals pooled per dissociation; 5 wells, 3–5 neurons per image) and 100 nM PTX-treated (teal, *n* = 14 animals, 3 independent DRG cell dissociations; 7 wells, 3–5 neurons per image) DRG neuron cultures**. (B)** Total neurite length (μm), **(C)** Mean neurite length (μm), **(D)** Complexity, **(E)** Number of nodes, **(F)** Number of neurites, **(G)** Number of neurite terminals. Data are normalised to baseline (*t* = 0 min). Statistical analysis was performed using two-way ANOVAs with multiple comparisons. Data are presented as violin plots with median lines. *p < 0.05, **p < 0.01. (For interpretation of the references to colour in this figure legend, the reader is referred to the web version of this article.)

It is well established that PTX inhibits the outgrowth of DRG sensory neurons in vitro; however, previous studies have relied solely on endpoint imaging to assess this effect. In this experiment, we used live-cell imaging to investigate the real-time, dynamic effects of PTX on DRG neurite outgrowth. This approach enabled a more detailed temporal analysis of neurite development, including measurements of total neurite and mean neurite length, complexity, and number of neurites, nodes, and terminals. Representative images of vehicle- and PTX-treated neurons are shown in Fig. 7A.

We found that PTX-treated neurons had significantly reduced total neurite length (*F* (1,10) = 31.91, *p* = 0.0002; Fig. 7B), mean neurite length (*F* (1, 10) = 17.75, *p* = 0.0018; Fig. 7C), complexity (*F* (1, 10) = 6.096, *p* = 0.0332; Fig. 7D), number of nodes (F (1, 10) = 10.75, *p* = 0.0083; Fig. 7E), and number of terminals (*F* (1, 10) = 14.14, *p* = 0.0037; Fig. 7G) compared to controls, with no significant effect found for number of neurites (*F* (1, 10) = 0.1562, *p* = 0.7010; Fig. 7F). Although no main effects of time were observed, significant time x treatment interactions were found for total neurite length (*F* (3,30) = 5.470, *p* = 0.0040; Fig. 7B), mean neurite length (*F* (3, 30) = 3.246, *p* = 0.0356; Fig. 7C), complexity (*F* (3, 30) = 4.941, *p* = 0.0066; Fig. 7D), and number of terminals (*F* (3, 30) = 3.767, *p* = 0.0209; Fig. 7G).

Multiple-comparison testing found that these effects were driven by PTX-treated neurons, which exhibited significantly reduced total neurite length at 200 min (*p* = 0.0343), 400 min (*p* = 0.0014) and 800 min (*p* = 0.0012) compared to vehicle-treated controls (Fig. 7B). Mean neurite length was also significantly decreased at 400 min (*p* = 0.013; Fig. 7C). Similarly, PTX significantly reduced the number of neurite nodes at 600 min (*p* = 0.0199) and 800 min (*p* = 0.0151; Fig. 7E), and the number of neurite terminals at 600 min (*p* = 0.0152) and 800 min (*p* = 0.0069; Fig. 7G).

Overall, these results demonstrate that PTX significantly inhibits multiple parameters of DRG neurite outgrowth, with significant effects emerging ∼200 min after treatment.

### 3.4 Tregs protect against PTX-induced inhibition of DRG sensory neuron outgrowth

**Fig. 8:**
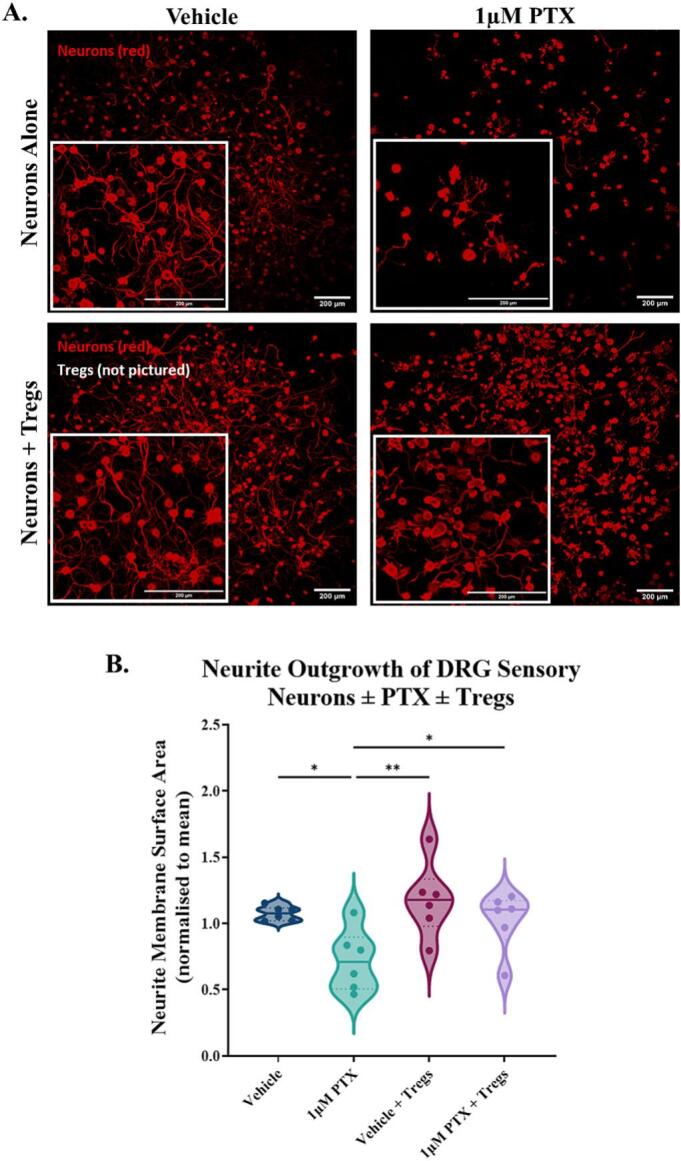
**Tregs protect against PTX-induced inhibition of DRG neurite outgrowth. (A)** Representative images of DRG sensory neurons cultured with or without Tregs and treated with vehicle or 1 μM PTX. Neurons were immunostained for β-III-tubulin (AF647, red). Tregs are not visible due to their non-adherent nature and loss during washes in the staining process. Scale bar represents 200 μm. **(B)** Fold change in neurite membrane surface area (μm^2^)^,^ relative to the mean value of its respective biological replicate of DRG neuron cultures under four conditions: neurons treated with vehicle alone (blue, *n* = 6 animals from 3 independent DRG cell dissociations with 2 animals pooled per dissociation; 6 wells), neurons treated with 1 μM PTX alone (teal, *n* = 6 animals from 3 independent DRG cell dissociations with 2 animals pooled per dissociation; 6 wells), vehicle-treated neurons co-cultured with Tregs (magenta, *n* = 12 animals from 3 independent DRG cell dissociations and 3 independent Treg dissociations with 2 animals pooled per dissociation; 6 wells), and PTX-treated neurons co-cultured with Tregs (purple, *n* = 12 animals from 3 independent DRG cell dissociations and 3 independent Treg dissociations with 2 animals pooled per dissociation; 6 wells). Statistical analysis was performed using one-way ANOVAs with multiple comparisons. Data are presented as violin plots with median and quartile lines. *p < 0.05, **p < 0.01. (For interpretation of the references to colour in this figure legend, the reader is referred to the web version of this article.)

To assess the neuroprotective effect of Tregs against PTX-induced inhibition of neurite outgrowth, Tregs were co-cultured with PTX- or vehicle-treated DRG sensory neurons; DRG neurons were cultured for 24 h and then treated with 1 μM PTX or vehicle for a further 24 h. After removal of treatment media, neurons were incubated with or without Tregs in complete neuronal medium for an additional 12 h. Neurite outgrowth was quantified by measuring neurite membrane surface area following immunostaining with anti-β-III tubulin. Representative images are provided in Fig. 8A.

One-way ANOVA revealed a significant effect of treatment condition on neurite outgrowth (*F* (2.545, 12.72) = 11.29, *p* = 0.0009; Fig. 8B). Tukey's post hoc test found that PTX significantly reduced neurite outgrowth compared to both vehicle-treated neurons (*p* = 0.0415) and vehicle-treated neuron + Treg co-cultures (*p* = 0.049). Importantly, co-culturing PTX-treated neurons with Tregs significantly rescued neurite outgrowth compared with PTX alone (*p* = 0.0178), restoring it to levels comparable to those of vehicle-treated neurons (*p* = 0.9370). These results demonstrate that Tregs protect DRG sensory neurons from PTX-induced inhibition of neurite outgrowth.

### 3.5 AG1478 blocks the neuritogenic effects of Tregs, while AG1478 and BIBO3304 inhibit Treg-mediated neuroprotection in PTX-treated DRG sensory neurons

Treg-mediators AREG and NPY are known to support neurite outgrowth (Hökfelt et al., 2008; Nilsson and Kanje, 2005) and exert neuroprotective effects (Ito et al., 2019a, Ito et al., 2019b; Li et al., 2019). To investigate whether these factors mediate the neuritogenic and neuroprotective effects of Tregs on DRG sensory neurons, their respective receptors – epidermal growth factor receptor (EGFR; the receptor for AREG) and NPY1 receptor (NPYR) – were blocked using AG1478 and BIBO3304, respectively.

**Fig. 9:**
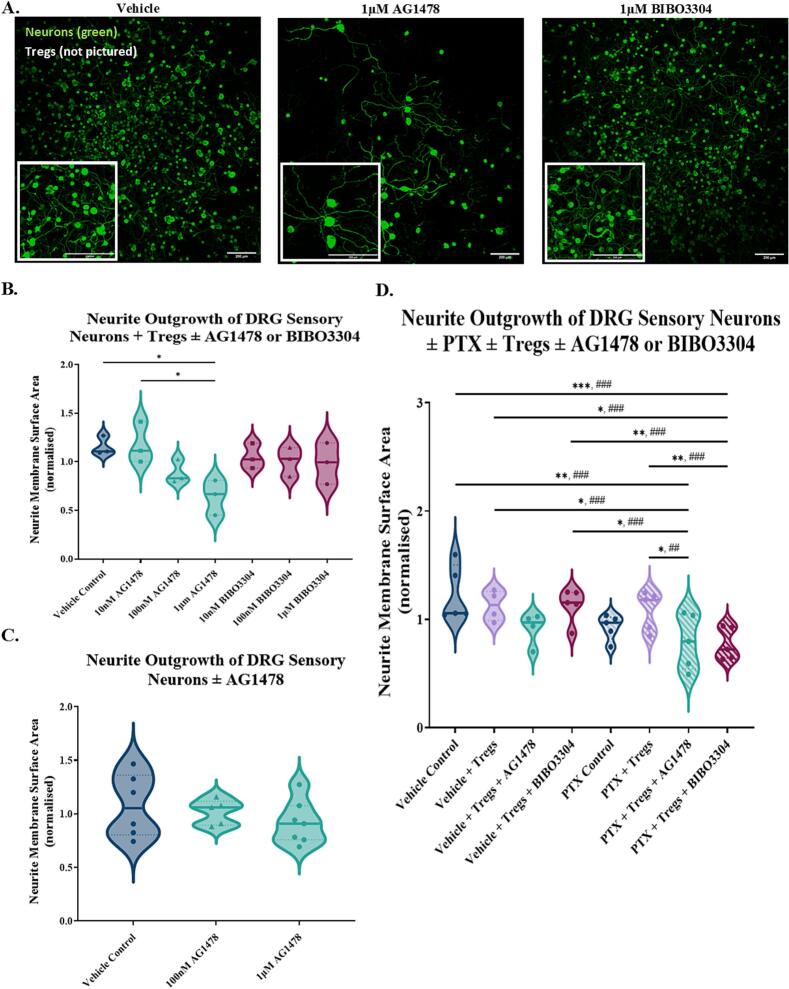
**Blocking AREG and NPY1 receptors prevents the neuroitogenic and neuroprotective effects of Tregs on DRG neurite outgrowth. (A)** Representative images of DRG sensory neurons co-cultured with Tregs and treated with vehicle (left), 1 μM AG1478 (middle), and 1 μM BIBO3304 (right). Neurons were stained for β-III tubulin (AF488, green). Tregs are not visible due to their non-adherent nature and their loss during immunostaining washes. Scale bar represents 200 μm. **(B)** Fold change in neurite membrane surface area (μm^2^)^,^ relative to the mean value of its respective biological replicate of DRG neurons co-cultured with Tregs and treated with vehicle (blue), or increasing concentrations of AG1478 (10 nM, 100 nM, 1 μM; teal) or BIBO3304 (10 nM, 100 nM, 1 μM, magenta). Shapes denote concentration: squares = 10 nM, triangles = 100 nM, circles = 1 μM. *n* = 12 animals from 3 independent DRG cell dissociations and 3 independent Treg dissociations with 2 animals pooled per dissociation; 3 wells. **(C)** Fold change in neurite membrane surface area (μm^2^)^,^ relative to the mean value of its respective biological replicate of DRG neurons cultured without Tregs and treated with vehicle (blue), 100 nM AG1478 (teal triangles), or 1 μM AG1478 (teal circles). *n* = 6 animals from 3 independent DRG cell dissociations with 2 animals pooled per dissociation; 5–7 wells. **(D)** Fold change in neurite membrane surface area (μm^2^)^,^ relative to the mean value of its respective biological replicate of DRG neurons cultures under eight conditions: vehicle control (blue), vehicle + Tregs (purple), vehicle + Tregs +1 μM AG1478 (teal), vehicle + Tregs +1 μM BIBO3304 (magenta), PTX control (blue, patterned), PTX + Tregs (purple, patterned), PTX + Tregs +1 μM AG1478 (teal, patterned), and PTX + Tregs +1 μM BIBO3304 (magenta, patterned). Only comparisons with PTX + Tregs + AG1478 and PTX + Tregs + BIBO3304 are presented. *n* = 12 animals from 3 independent DRG cell dissociations and 3 independent Treg dissociations with 2 animals pooled per dissociation; 4–5 wells. Statistical analysis was performed using one-way ANOVAs with multiple comparisons corrected for false discovery rate. Data are presented as violin plots with median and quartile lines. *p < 0.05, **p < 0.01, ***p < 0.001, ## large effect size (0.8 < *g* < 1.2), ### very large effect size (*g* > 1.2). (For interpretation of the references to colour in this figure legend, the reader is referred to the web version of this article.)

DRG neuron + Treg co-cultures were treated with increasing concentrations of AG1478 or BIBO3304 (10 nM, 100 nM and 1 μM). Representative images are shown in Fig. 9A. One-way ANOVA revealed a dose-dependent reduction in neurite outgrowth with AG1478 (*F* (3, 8) = 3.715, *p* = 0.0202; Fig. 9B), with significant inhibition at 1 μM compared to vehicle (adj. *p* = 0.0174) and 10 nM AG1478 (adj. *p* = 0.0174). BIBO3304 had no significant effect (Fig. 9B). To determine whether AREG may act in an autocrine manner, AG1478 was applied to DRG neurons alone, but no effect on outgrowth was observed (Fig. 9C), suggesting that AREG in this context was Treg-derived.

Next, the role of AREG and NPY in Treg-mediated neuroprotection against PTX was assessed. Neurons were treated with PTX and co-cultured with Tregs in the presence or absence of AG1478 or BIBO3304. One-way ANOVA revealed a significant effect of treatment condition on neurite outgrowth (*F* (7, 30) = 4.056, *p* = 0.0031; Fig. 9D). Consistent with previous experiments, PTX significantly reduced neurite outgrowth (adj. *p* = 0.0449, *g* = −1.37 [95% CI -2.35, −0.784]) and co-culture with Tregs restored outgrowth relative to vehicle controls (adj. *p* = 0.2830, *g* = −0.605 [95% CI -1.79, 0.77]). Blocking EGFR with AG1478 again significantly inhibited outgrowth in vehicle + Treg co-cultures compared to vehicle alone (adj. *p* = 0.0455, *g* = −1.28 [95% CI -2.29, −0.724]), whereas BIBO3304 had minimal impact (adj. *p* = 0.4282, *g* = 0.418 [95% CI -1.59, 1.0]).

Importantly, both AG1478 and BIBO3304 significantly prevented Treg-mediated neuroprotection in PTX-treated neurons with very large effect sizes. PTX + Tregs + AG1478 significantly reduced outgrowth compared to vehicle alone (adj. *p* = 0.0096, *g* = −1.53 [95% CI -2.8, −0.64]), vehicle + Tregs (adj. *p* = 0.0449, *g* = −1.37 [95% CI -2.88, −0.32]), vehicle + Tregs + BIBO3304 (adj. *p* = 0.0384, *g* = −1.44 [95% CI -2.87. -0.0979]), and PTX + Tregs (adj. *p* = 0.0490, *g* = −1.16 [95% CI -2.77, 0.0391]). Similarly, PTX + Tregs + BIBO3304 significantly reduced outgrowth compared to vehicle (adj. *p* = 0.0096, *g* = −1.97 [95% CI -2.85, −1.28]), vehicle + Tregs (adj. *p* = 0.0384, *g* = −2.15 [95% CI -3.84, −1.15]), vehicle + Tregs + BIBO3304 (adj. *p* = 0.0363, *g* = −2.13 [95% CI -4.41, −0.667]) and PTX + Tregs (adj. *p* = 0.0449, *g* = −1.66 [95% CI -3.6, −0.39]).

Together, these results demonstrate that both AREG and NPY contribute to Treg-mediated neuroprotection in PTX-treated neurons. Blocking either receptor impairs Treg-mediated rescue of neurite outgrowth, suggesting that these pathways are key effectors of Treg neuroprotection.

## 4 Discussion

In this study, we demonstrate that Tregs directly promote DRG sensory neuron outgrowth and confer protection against PTX-induced neurotoxicity in vitro. Activated Tregs markedly enhanced neurite outgrowth, increasing neurite length and branching, and preferentially localised to neuron-rich regions and neurite terminals, indicating active and spatially targeted Treg-neuron interactions. Consistent with previous reports, PTX exposure significantly impaired neurite outgrowth; however, co-culture with activated Tregs reversed this deficit, restoring neurite outgrowth to levels comparable to those of untreated neurons. Mechanistically, pharmacological inhibition identified AREG as a critical mediator of Treg-induced neurite outgrowth, as blockade of AREG signalling abolished these growth-promoting effects. In contrast, both AREG and NPY signalling were required for Treg-mediated neuroprotection following PTX exposure, as antagonism of either pathway significantly impaired recovery of neurite outgrowth. Together, these findings identify Tregs as active modulators of peripheral sensory neurons and reveal distinct molecular pathways underlying their growth-promoting and neuroprotective functions.

Although research on Treg-DRG sensory neuron interactions remains limited, emerging research suggests that Tregs directly modulate sensory neuron activity and contribute to pain relief by releasing neuropeptides, such as enkephalin, an endogenous opioid (Aubert et al., 2024; Mendoza et al., 2025; Midavaine et al., 2025). Experimental depletion of enkephalin-producing Tregs increases DRG neuronal activation and pain sensitivity, independent of their immunosuppressive functions, suggesting a direct neuroimmune mechanism (Mendoza et al., 2025). Both skin-resident and meningeal Tregs have been shown to suppress excessive sensory neuron excitability through opioid signalling (Mendoza et al., 2025; Shime et al., 2020). In particular, expansion of meningeal Tregs reduces spared nerve injury-induced pain in a sex-dependent manner (Midavaine et al., 2025). Beyond DRG sensory neurons, Tregs interact with diverse neuronal subtypes. In models of Parkinson's disease, Tregs protect dopaminergic neurons via contact-dependent mechanisms (Huang et al., 2017, Huang et al., 2020). Peripheral sympathetic neurons from the superior cervical ganglion can induce FoxP3 expression in CD4^+^ T cells through transforming growth factor-beta (TGF-β), IL-10 and calcitonin gene-related peptide (CGRP) signalling, an effect that requires direct contact and is not recapitulated by conditioned media alone (Szklany et al., 2016). Similarly, cortical neurons induce Treg differentiation from encephalitogenic T cells via CD28- and TGF-β-dependent contact, resulting in suppression of experimental autoimmune encephalomyelitis (EAE) (Liu et al., 2006). Conversely, superior cervical ganglion neurons survive in co-culture with T cells in the absence of nerve growth factor, indicating reciprocal trophic support (Szklany et al., 2016). Together, these studies support bidirectional neuron–Treg interactions, consistent with our findings. Here, we observed that activated Tregs were enriched in culture regions containing DRG neurons and preferentially localised to neurite terminals, indicating targeted Treg–neuron interactions. Although the underlying mechanisms remain unclear, reciprocal chemoattractive signalling between neurons and Tregs is likely to promote this spatial proximity and facilitate functional crosstalk, enhancing neurite outgrowth and local Treg accumulation.

Tregs may influence neuronal outgrowth through paracrine signalling by secreting immunomodulatory and trophic mediators that act directly on neurons, including AREG and NPY. AREG, a ligand of the EGFR, is highly expressed by tissue-resident Tregs, particularly those residing in the nervous system, skin, and skeletal muscle (Liston et al., 2022; Shime et al., 2020; Zhao et al., 2017). In DRG neurons, AREG has been shown to significantly enhance axonal outgrowth in both organotypic and dissociated cultures, an effect blocked by the EGFR inhibitor AG1478 (Nilsson and Kanje, 2005). Consistently, we found that AG1478 dose-dependently inhibited Treg-facilitated neurite outgrowth in the co-cultures. As AREG can also be produced by other cell types, including DRG neurons themselves, we tested whether AG1478 would reduce outgrowth in neurons cultured alone. No significant effect was observed, indicating that the facilitation of neurite outgrowth in co-cultures is specifically mediated by Treg-derived Areg. These findings suggest that Tregs promote neurite outgrowth, at least in part, through paracrine AREG-EGFR signalling.

NPY is a neuromodulatory peptide that functions in both the central and peripheral nervous systems in regions critical to pain. Although expressed at low levels in DRG neuron cell bodies, its receptors, NPY1 and NPY2, are present on their axons (Brumovsky et al., 2002). Exogenous NPY has been shown to enhance neurite outgrowth of dissociated DRG neurons co-cultured with spinal cord slices (White and Mansfield, 1996) and increase multiple metrics of neurite growth, including total length, number of neurites, and branching complexity (Ring et al., 2006). Additionally, NPY has been reported to influence the growth rate and angle of prenatal DRG neurons via NPY1 receptor signalling (Hökfelt et al., 2008). However, inhibiting NPY signalling with the NPY1 antagonist, BIBO3304, did not block the Tregs' neuritogenic effect, which may be due to low basal expression levels of NPY1 receptors in uninjured adult DRG sensory neurons (Brumovsky et al., 2004).

In addition to AREG and NPY, Treg-derived cytokines such as IL-10 and TGF-β may contribute to neurite outgrowth by regulating cytoskeletal dynamics (Ye et al., 2022; Zhou et al., 2009). Tregs can also express or induce neurotrophic factors, including brain-derived neurotrophic factor (BDNF), particularly in inflammatory or tissue repair contexts (Ip and Wischhusen, 2023). BDNF-TrkB signalling promotes growth cone sprouting, cytoskeletal remodelling, and neurite extension (McGregor and English, 2019). Conversely, DRG neurons release a range of mediators that can influence Treg recruitment and function (Gao et al., 2022). In the gut, prolonged activation of TRPV1^+^ sensory neurons alters Treg transcriptomes and proliferation via CGRP signalling. Enteric neurons modulate Treg induction through soluble, IL-6-dependent mechanisms in vitro, highlighting a neuroimmune feedback loop in which neuronal activity can shape Treg behaviour (Yan et al., 2021). Collectively, these findings demonstrate complex bidirectional interactions between Tregs and sensory neurons and suggest that various Treg-secreted mediators may promote DRG neurite outgrowth. Although Tregs closely associate with and appear attracted to DRG neurites, the molecular and contact-dependent mechanisms governing this interaction remain to be determined.

Accumulating evidence from human and animal studies indicates that Tregs exert important neuroprotective functions across an array of neurological conditions and injuries (Chen et al., 2022; Davoli-Ferreira et al., 2020; Dombrowski et al., 2017; Duffy et al., 2018; Fiore et al., 2023; Heyn et al., 2016; Luchting et al., 2015; Mendoza et al., 2025; Milling and Edgar, 2019; Raposo et al., 2014; Sakaguchi et al., 2020; Wang et al., 2015; Xing et al., 2013; Zhang et al., 2024). Using advanced live-cell and confocal imaging, we examined the neuroprotective capacity of Tregs in an in vitro model of PTX-induced neurotoxicity. High-resolution live imaging with ZEISS Lattice Lightsheet 7 enabled real-time characterisation of PTX-induced neuronal injury, revealing rapid and sustained reductions in neurite length, branching complexity, and terminal formation, with significant inhibition of total neurite outgrowth observed as early as 200 min after PTX exposure. Unlike previous studies relying on fixed endpoint analyses (Chen et al., 2017; Gornstein and Schwarz, 2017; Livni et al., 2022; Pani et al., 2014; Ustinova et al., 2013), live-cell imaging captured the rapid and progressive impact of PTX-induced changes in DRG neuron morphology, and is consistent with established reports of PTX-induced inhibition of neuron outgrowth (Chen et al., 2015; Kawashiri et al., 2018; Livni et al., 2019, Livni et al., 2022; Villalba-Riquelme et al., 2022).

We next assessed whether Tregs mitigate PTX-induced inhibition of DRG neuron outgrowth. Co-culture with Tregs restored neurite outgrowth in PTX-treated neurons to the level of vehicle-treated neurons. This finding aligns with previous work reporting neuroprotective and analgesic roles for Tregs in models of PNI and EAE (Austin et al., 2012; Davoli-Ferreira et al., 2020; Duffy et al., 2019; Midavaine et al., 2025), as well as in CIPN, where adoptive transfer of Tregs significantly alleviated mechanical allodynia in PTX-treated mice (Liu et al., 2014). Recent studies have begun to elucidate the mechanisms of peripheral Treg-mediated analgesia. In murine models of PNI, Tregs infiltrate and proliferate at the injury site (Davoli-Ferreira et al., 2020), and their expansion via adoptive transfer and IL-2/anti-IL-2 immunocomplex treatment significantly attenuated mechanical allodynia and suppressed neuroinflammation through IL-10-dependent inhibition of CD4^+^ T helper 1 responses (Davoli-Ferreira et al., 2020). Tregs also promote M2-like macrophage polarisation in the DRG, enhancing axonal regeneration and reducing mechanical hypersensitivity following sciatic nerve ligation (Chen et al., 2022). Beyond immunomodulation, Tregs directly regulate peripheral sensory neuron activity through endogenous opioid production, further contributing to their analgesic effects (Mendoza et al., 2025; Shime et al., 2020). These studies support a role for Tregs in neural repair and neuroprotection, consistent with our findings.

Treg neuroprotective effects were abolished when the co-cultures were treated with AG1478 or BIBO3304, suggesting that AREG and NPY are both involved in this mechanism. This is in line with previous research reporting the neuroprotective functions of these mediators. For example, Tregs accumulate in the brain after ischemic stroke, where they contribute to neurological recovery (Li et al., 2013; Wang et al., 2015; Xu et al., 2023), a process in which AREG was identified as a key mediator (Ito et al., 2019a). In the context of PNI, AREG has similarly been implicated in supporting nerve repair. Elevated AREG levels promote Schwann cell proliferation and migration, facilitating neurite outgrowth and axonal regeneration (Chen et al., 2023). Its upregulation in the DRG of TGF-α knockout mice following sciatic nerve injury further supports its involvement in neural survival and regeneration (Xian et al., 2001). However, emerging evidence suggests that AREG may also contribute to the persistence of neuropathic pain. Although AREG is upregulated in the DRG following PNI and supports repair, it can also enhance sensory neuronal excitability and pro-inflammatory gene expression through histone lactylation (Deng et al., 2025). This dual role highlights the context-dependent actions of Treg-derived AREG, with regenerative versus pronociceptive effects shaped by cellular environment, timing, and inflammatory cues.

Although direct evidence for a protective role of Treg-derived NPY is limited, extensive studies implicate NPY in nerve injury responses and neuroprotection. Following sciatic nerve injury, NPY is significantly upregulated in the ipsilateral DRG neurons (Verge et al., 1995; Wakisaka et al., 1991) and is thought to be an adaptive response to hyperalgesia-induced excitatory signalling (Diaz-delCastillo et al., 2018). Intrathecal NPY reduces nociceptive reflexes and hyperalgesia in nerve injury and inflammatory pain models (Intondi et al., 2008; Taiwo and Taylor, 2002; Xu et al., 1994, Xu et al., 1998) via NPY1 receptors, as these effects were abolished by BIBO3304 (Intondi et al., 2008; Taiwo and Taylor, 2002). NPY1 receptor expression is also upregulated in the spinal dorsal horn during inflammatory pain, where NPY inhibits substance P release and reduces hyperalgesia (Taylor et al., 2014).

Peripheral NPY administration, however, exhibits context-dependent effects, showing both pro- and anti-nociceptive actions on pain modality; for instance, subcutaneous injection of an NPY1 agonist increases mechanical but reverses thermal hyperalgesia (Tracey et al., 1995). Centrally, NPY acts on NPY1 receptors in the spinal cord to reduce neuropathic and inflammatory pain, and can mask latent pain sensitisation (Nelson et al., 2022; Nie and Taylor, 2025). NPY is also produced by diverse neuronal and immune cell populations (Chen et al., 2020; Zhang et al., 2021). Our findings identify a previously unrecognised Treg-neuron interaction in which AREG and NPY contribute to neurite protection in vitro; however, these mediators are likely to represent only part of the broader signalling network underlying Treg-mediated neuroprotection. Further in vivo studies are required to define their contribution within the complex neuroimmune environment that regulates neuronal injury and repair.

There are potential caveats to the translation of the current findings. Neurite outgrowth requires tight regulation, as excessive or aberrant sprouting in the DRG and spinal dorsal horn is associated with neuropathic pain behaviours following nerve injury (Abbadie and Basbaum, 1998; Chien et al., 2005; Chung et al., 1996). Such maladaptive sprouting can drive aberrant sensory transmission and sensitisation by increasing neuronal excitability, spontaneous activity, and disrupting the local inflammatory environment (Chen and Zhang, 2015; Cohen and Mao, 2014; Ramer et al., 1999; Xie et al., 2006). Accordingly, Treg-driven neuritogenesis must be carefully balanced to promote targeted regeneration without exacerbating pain. Additionally, Tregs suppress anti-tumour immunity by inhibiting effector T cells and NK cells, and high intratumoural Treg abundance is associated with poor cancer prognosis (Curiel, 2008; Zou, 2006). Indeed, depleting Tregs enhances the efficacy of cancer immunotherapies (Kurose et al., 2015; Morse et al., 2008; Rech et al., 2012) and PTX itself reduces intratumoral Treg numbers, which contributes to its anti-tumour effect (Vicari et al., 2009; Yu et al., 2022). This presents a therapeutic dichotomy as Tregs appear to protect against CIPN, but systemic Treg expansion may compromise anti-cancer immunity, supporting the development of local or tissue-targeted Treg-based therapies.

### 4.1 Limitations of the study

Several limitations of this study should be acknowledged. First, the current study relies exclusively on in vitro models, which do not fully capture the systemic and cumulative nature of PTX exposure in vivo. Neurons were exposed to PTX before extensive neurite outgrowth was established, limiting the model's reflection of mature neuronal network dynamics. While prior work shows that enhancing Treg populations, such as through adoptive transfer, can alleviate neuropathic pain (Austin et al., 2012; Davoli-Ferreira et al., 2020; Duffy et al., 2019; Fischer et al., 2019; Lees et al., 2015; Zhang et al., 2024), it remains unclear how Tregs influence DRG neurite outgrowth in vivo. Future studies should explore whether adoptive Treg transfer in animal models of CIPN reproduces the neuroprotective effects observed here.

Second, although AREG and NPY emerged as candidate mediators of Treg-induced neurite outgrowth and neuroprotection, we did not directly quantify their secretion by Tregs under the culture conditions used in this study. Future studies should therefore determine the levels and kinetics of endogenous AREG and NPY release, as well as those of other mediators (e.g., cytokines, growth factors), and establish their relative contributions to Treg-mediated effects on sensory neurons.

Third, neurons and Tregs were derived exclusively from female mice. Although this choice has clinical relevance, given that paclitaxel is commonly used to treat cancers that disproportionately affect women, and that female sex is associated with a higher risk of developing high-grade CIPN (Alqahtani et al., 2019; Johnson et al., 2023; von Itzstein et al., 2025; Weaver, 2014), the extent to which the observed Treg-mediated effects generalise to males remains unknown. This is particularly relevant given emerging evidence for sex-dependent neuroimmune mechanisms in pain (Bethea and Fischer, 2021; Gregus et al., 2021; Mogil, 2012) and indications that Tregs may play a more prominent role in females (Kuhn et al., 2021; Midavaine et al., 2025). Future studies should therefore evaluate Treg-neuron interactions also in males to determine whether Treg-mediated neuroprotection exhibits sexual dimorphism.

### 4.2 Conclusions

In summary, we show that Tregs directly interact with DRG sensory neurons, promote neurite outgrowth, and confer protection against PTX-induced neurotoxicity, potentially via mediators such as AREG and NPY. These findings identify a previously unrecognised neuroimmune interaction that may contribute to sensory neuron repair and protection. However, direct Treg-neuron signalling is likely to represent only one component of the complex cellular and molecular mechanisms that regulate neuronal injury and recovery in vivo. While our observations are derived from in vitro cultures of female DRG neurons, they provide a foundation for future studies to determine whether Tregs or their mediators can promote recovery and protect sensory neurons in vivo following chemotherapy or other forms of neural injury. Future studies should also evaluate these mechanisms in males to assess potential sex-dependent differences in Treg-mediated neuroprotection.

The following are the supplementary data related to this article.

Supplementary Video S1 Representative time-lapse of a co-culture of DRG sensory neurons (green) and activated Tregs (red). DRG sensory neurons were grown for 24 hours before being co-cultured with Tregs and imaged every 2 hours for 24 hours on the ZEISS Lattice Lightsheet 7 microscope. Scale bar represents 100 µm.

Supplementary material 1

## Authorship contribution

Jessica Hayes and Gila Moalem-Taylor conceptualised the study. Jessica Hayes designed and performed the experiments, conducted image acquisition on the ZEISS LSM 710 microscope, carried out image and data analysis, and drafted the manuscript. Sandra Fok optimised ZEISS Lattice Lightsheet 7 imaging parameters and performed both image acquisition and analysis. Gary Housley provided guidance on confocal microscopy imaging and statistical analysis, while Gila Moalem-Taylor contributed intellectual input throughout the experimental and analytical phases. Renee Whan facilitated access to the pre-serial version of the ZEISS Lattice Lightsheet 7 upon its arrival at UNSW in 2021. All authors provided feedback and editorial support on the manuscript.

## Declaration of generative AI use

During the preparation of this work, the authors used Grammarly and ChatGPT (OpenAI) for language editing and text refinement. After using this tool, the authors reviewed and edited the content as needed and take full responsibility for the content of the published article.

## CRediT authorship contribution statement

**Jessica P. Hayes:** Writing – review & editing, Writing – original draft, Methodology, Investigation, Formal analysis, Data curation, Conceptualization. **Sandra Fok:** Writing – review & editing, Methodology, Formal analysis. **Renee Whan:** Resources, Methodology. **Gary D. Housley:** Writing – review & editing, Supervision, Resources, Methodology, Formal analysis. **Gila Moalem-Taylor:** Writing – review & editing, Supervision, Project administration, Funding acquisition, Conceptualization.

## Funding

This study was supported by a grant from the 10.13039/501100000925National Health and Medical Research Council of Australia (NHMRC) awarded to Gila Moalem-Taylor (ID # APP1162060).

## Declaration of competing interest

The authors declare that they have no known competing financial interests or personal relationships that could have appeared to influence the work reported in this paper.

## Data Availability

Data will be made available on request.
