# Supplementary material for: Regulatory T cells promote sensory neuron growth and protect against neurotoxicity
Source: Neurobiol Pain. 2026 Jun 30;20:100224. doi: 10.1016/j.ynpai.2026.100224 (PMC13382340; doi:10.1016/j.ynpai.2026.100224)
Supplement: Supplementary material 1 [file mmc2.pdf]

Hayes et al. Supplemental material

“*Regulatory T Cells Promote Sensory Neuron Growth and Protect Against Neurotoxicity*”

Supplementary Tables

Table S1 ZEISS Lattice Lightsheet 7 imaging parameters

Parameter	Dimension
Illumination objective lens	10x / 0.4, 30° angle to cover glass
Detection objective lens	48x / 1.0, 60° angle to cover glass
Camera	Pco.edge 4.2 CLHS sCMOS
Lattice lightsheet	Pre-defined Sinc3 beam with 30um length x 1,000 nm thickness
Filter	LBF 405 / 488 / 561 / 642
Detection wavelength	420-470, 503-546, 576-617, 656-750
Laser	0.3% 488nm diode 10mW power out (2mW in pupil)
Exposure time	120-400ms (depending on sample staining intensity)
Total scan time	24 hours
Duration	Stack scan every 120 minutes
Image size (pixels)	2048x700
Scaling (µm)	0.145 x 0.145 x 0.200
Scan interval (µm)	0.2
Bit Depth	16-bit
Sample scan (mm)	~1.55

Table S2 Quadrant condition criteria for Treg density analysis

Condition	Quadrant Criteria
(1) Containing neurons	• Contained neuronal somas<br>• Contained neurites that traverse the entire quadrant
(2) Not containing neurons	• Contained only a partial entrance of a neurite<br>• Did not contain neuronal somas or neurites<br>• Contained Tregs
(3) Excluded from analysis	• Did not contain neuronal somas, neurites or Tregs

Supplementary Figures

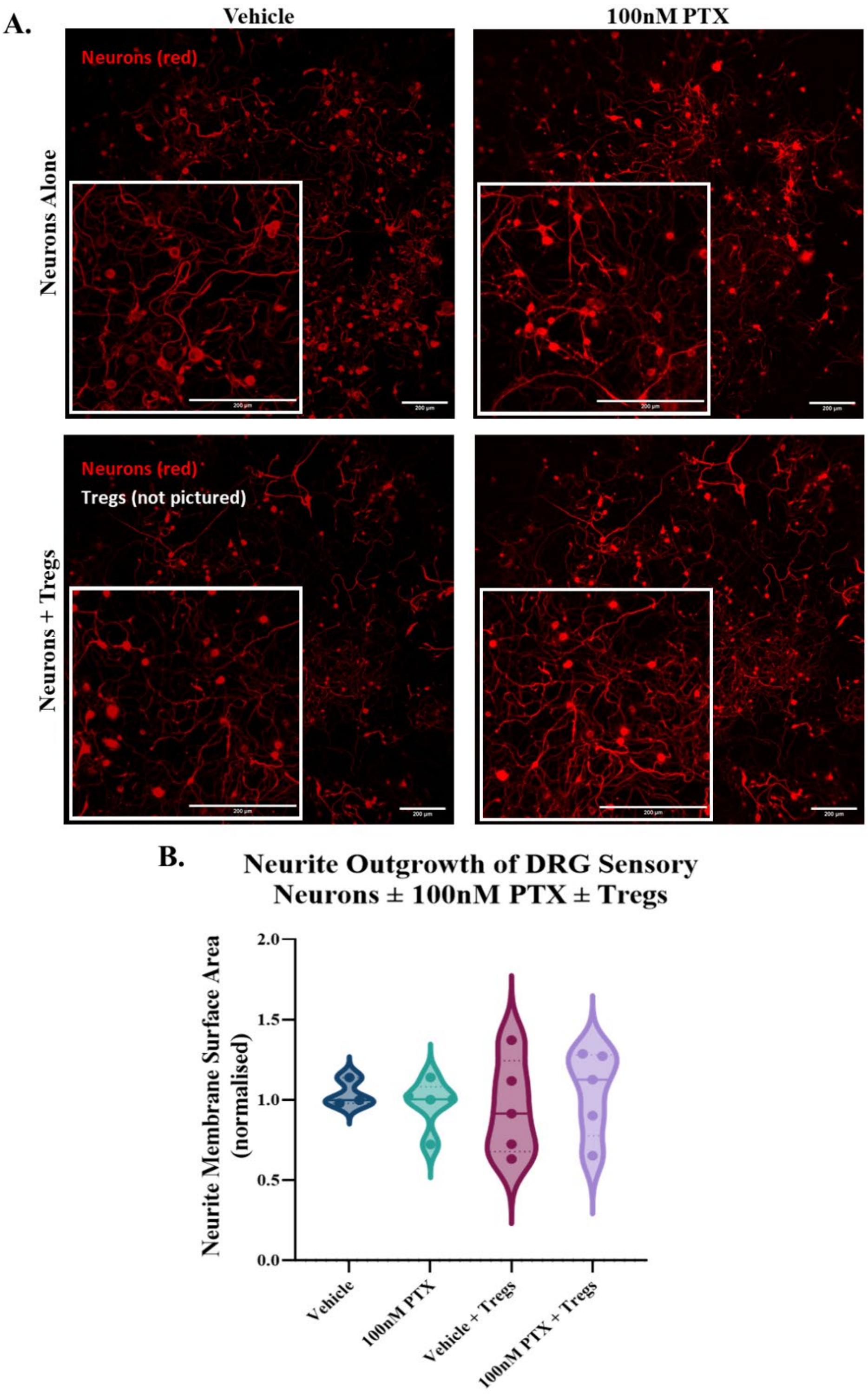

Fig. S1. Neurite outgrowth of DRG sensory neurons treated with vehicle or 100 nM PTX and co-cultured with Tregs for 24 hours. (A) Representative images of DRG sensory neurons cultured with or without Tregs and treated with vehicle or 100 nM PTX. Neurons were stained for β-III-tubulin (AF647, red). Tregs are not visible due to their non-adherent nature and their loss during

immunostaining washes. Scale bars represent 200 µm. **(B)** Neurite outgrowth of DRG sensory neurons under the following conditions: neurons + vehicle (navy, n = 6 animals from 3 independent DRG cell dissociations with 2 animals pooled per dissociation; 3 wells), neurons + 100 nM PTX (teal, n = 6 animals from 3 independent DRG cell dissociations with 2 animals pooled per dissociations; 5 wells), neurons + vehicle + Tregs (magenta, n = 12 animals from 3 independent DRG cell dissociations and 3 independent Treg dissociations with 2 animals pooled per dissociation; 5 wells), and neurons + 100 nM PTX + Tregs (purple, n = 12 animals from 3 independent DRG cell dissociations and 3 independent Treg dissociations with 2 animals pooled per dissociation; 5 wells). Statistical analysis was performed using one-way ANOVAs with multiple comparisons. Data are presented as violin plots with median and quartile lines.

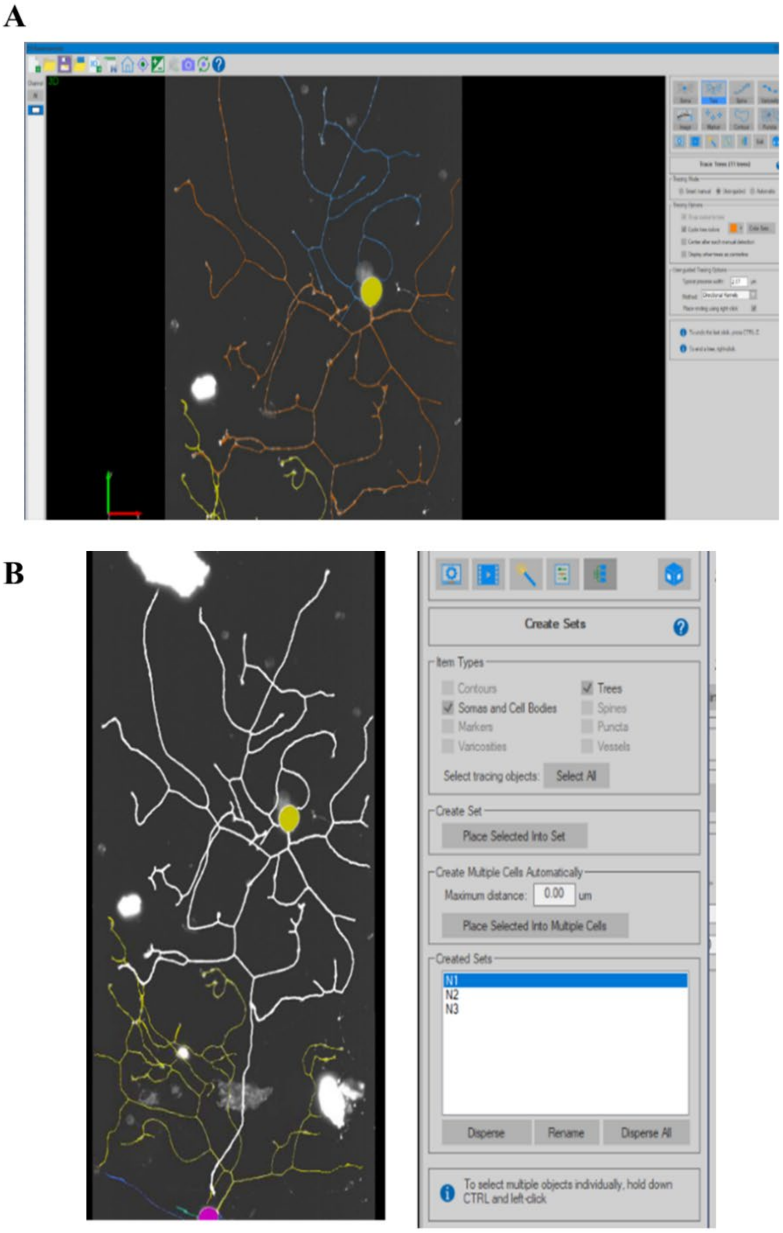

Fig. S2. Neuron tracing using NeuroLucida 360. (A) Screenshot of the NeuroLucida 360 3D Environment window showing a representative neuron with the soma (yellow) and neurites (orange and blue) traced. The soma was traced using the *AutoContour* function in the NeuroLucida main window, while neurites were traced using the semi-automated *User-Guided Trace Tree* function in the 3D Environment. **(B)** The same neuron was selected, and its soma and neurites ("somas and cell bodies" and "trees") were added to a *SET* in the 3D Environment to enable batch analysis of multiple neurons within a single image using NeuroLucida Explorer.

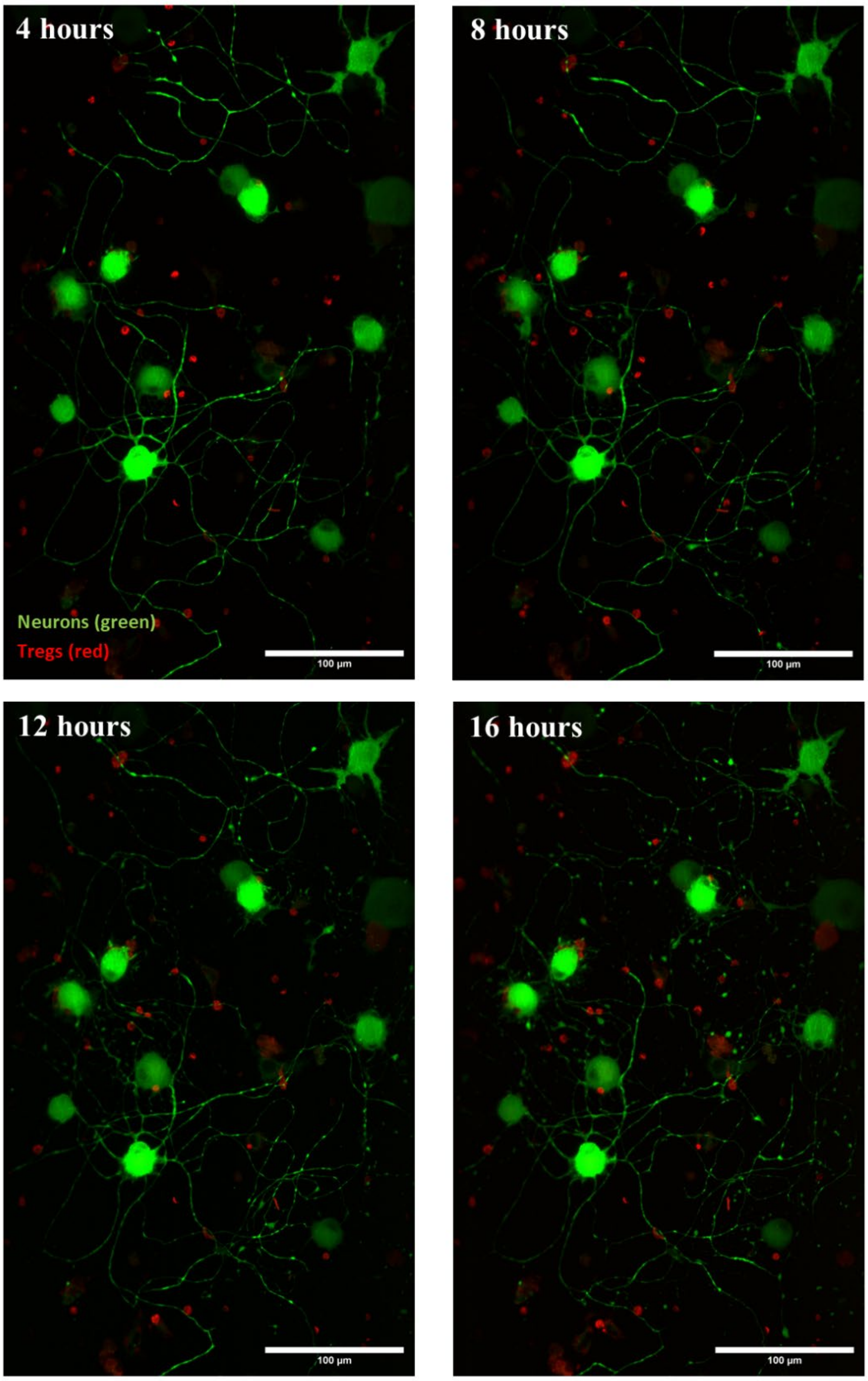

Fig. S3. Representative images of DRG sensory neuron and resting Treg co-culture at 4-, 8-, 12- and 16-hour time points. DRG sensory neurons were grown for 24 hours before being co-cultured with resting Tregs and imaged every 2 hours for 24 hours on the ZEISS Lattice Lightsheet 7 microscope. Neurons are dyed with NeuO dye (green), and Tregs are dyed with CellMask^{TM} deep red (red). Scale bar represents 100µm.

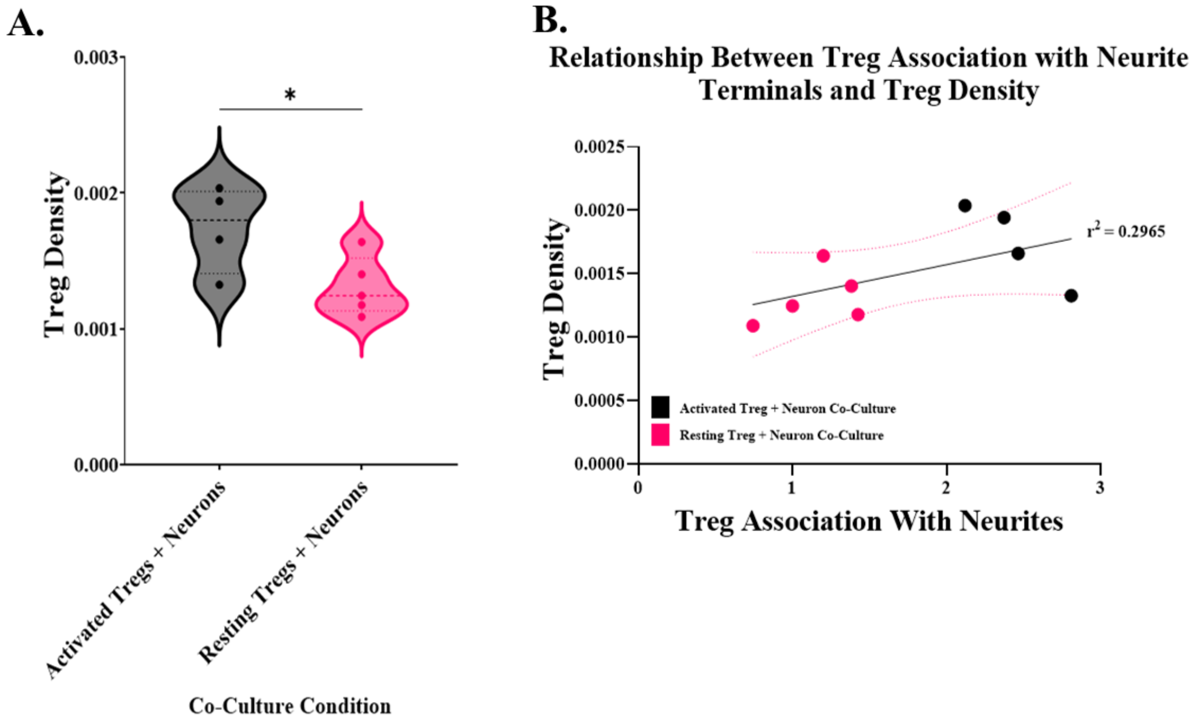

Figure S4. Activated Treg–DRG neurite association is independent of proliferation. Treg proliferation does not solely account for the greater association between activated Tregs and DRG sensory neuron terminals compared to resting Tregs and DRG sensory neuron terminals. **(A)** Density of Tregs in each image at 16 hours from the activated Tregs + neurons (black) and resting Tregs + neurons (pink) co-cultures. As image sizes varied, the density rather than the number of Tregs was calculated. Each point within the violin plot represents a different image. An unpaired two-tailed *t*-test found that there was significantly greater density of Tregs in the activated Treg + neuron co-culture condition compared to the resting Treg + neuron condition. $^{*}p < 0.05$, n = 4-5 cultures (derived from n = 12 animals from 3 independent DRG cell dissociations and 3 independent Treg dissociations with 2 animals pooled per dissociation) at 16 hours. **(B)** Relationship between Treg association with DRG sensory neuron terminals (number of Tregs per neurite) and Treg density found using a simple linear regression model. Pink dotted lines represent 95% CI. $r^2 = 0.2965$, n = 9 images.
